# Posttranslational control of lipogenesis in the tumor microenvironment

**DOI:** 10.1186/s13045-022-01340-1

**Published:** 2022-08-29

**Authors:** Yahui Zhu, Xingrong Lin, Xiaojun Zhou, Edward V. Prochownik, Fubing Wang, Youjun Li

**Affiliations:** 1 Hubei Key Laboratory of Cell Homeostasis, College of Life Sciences, TaiKang Center for Life and Medical Sciences, Wuhan University, Wuhan, 430072 China; 2 Frontier Science Center for Immunology and Metabolism, Medical Research Institute, Wuhan University, Wuhan, 430071 China; 3 School of Medicine, Chongqing University, Chongqing, 400030 China; 4 Division of Hematology/Oncology, Children’s Hospital of Pittsburgh of UPMC, The Department of Microbiology and Molecular Genetics, The Pittsburgh Liver Research Center and The Hillman Cancer Center of UPMC, The University of Pittsburgh Medical Center, Pittsburgh, PA 15224 USA; 5 Department of Laboratory Medicine, Zhongnan Hospital of Wuhan University, Wuhan, 430072 China

**Keywords:** Posttranslational modification, Cancer, Tumor microenvironment, Lipid metabolism reprogramming

## Abstract

Metabolic reprogramming of cancer cells within the tumor microenvironment typically occurs in response to increased nutritional, translation and proliferative demands. Altered lipid metabolism is a marker of tumor progression that is frequently observed in aggressive tumors with poor prognosis. Underlying these abnormal metabolic behaviors are posttranslational modifications (PTMs) of lipid metabolism-related enzymes and other factors that can impact their activity and/or subcellular localization. This review focuses on the roles of these PTMs and specifically on how they permit the re-wiring of cancer lipid metabolism, particularly within the context of the tumor microenvironment.

## Introduction

Over the past few years, it has been increasingly appreciated that enhanced macromolecular biosynthesis, altered energy metabolism and maintenance of REDOX homeostasis are fundamental features of cancer [1]. The metabolic changes that accompany tumor cells have attracted particular attention, especially with regard to how such changes could be harnessed for therapeutic benefit [2]. Lipid metabolic reprogramming is one such recently appreciated marker of tumor malignancy that has attracted considerable attention [3].

Molecularly, lipids are highly heterogeneous and, as a group, are comprised of thousands of different types of triglycerides, phospholipids, sphingolipids, glycolipids, cholesterol and cholesterol esters [4]. Intracellular lipids levels reflect a dynamic balance among their highly variable rates of biosynthesis, uptake, output and esterification, with excesses being secreted as lipoproteins and/or stored in lipid droplets [5]. An example of lipid metabolism reprogramming can occur in cancer-associated fibroblasts, where it promotes colorectal cancer cell metastasis in vitro and in vivo [6]. Similar, but deliberate reprogramming of lipid metabolism by tumor-associated T cell represents a new strategy for therapeutic immunometabolic modulation [7].

Cancer cells increase the uptake of preformed lipids from external sources in response to changes in environmental conditions. Meanwhile, these cells also activate de novo synthesis of fatty acids and transcriptional regulators of lipid biosynthesis are well-known positive targets of oncogenes and negative targets of tumor suppressor pathways [8–10]. Therefore, various lipid-metabolizing and lipid-modifying enzymes are potentially high-value candidates for therapeutic targeting in cancer [11]. Several recent reviews have summarized the multiple functions of lipid metabolism reprogramming in cancers, cancer-associated fibroblasts and other cells residing in the tumor microenvironment generally with an eye toward targeting these altered pathways as potential therapeutic options [12–14]. However, the precise function of the posttranslational modification of lipid metabolic enzyme has never reviewed in cancer and tumor microenvironment.

## Overview of lipid metabolism

In mammalian cells, lipids can be made available both through de novo synthesis and the external uptake and transport of preformed lipids via lysosomal–peroxisomal–ER pathways [15]. Most such cells take up cholesterol from low-density lipoproteins (LDLs) through receptor-mediated endocytosis. Upon reaching the lysosome, cholesterol is transported via NPC1/2 to the ER and other downstream organelles, to meet specific structural and functional needs. Peroxisomes also obtain cholesterol from the lysosome through direct lysosomal–peroxisome membrane contact. Peroxisomes directly engage the ER via peroxisomal PI(4,5)P2 and ER-resident extended synaptotagmin-1, 2 and 3 (e-syts). Cholesterol is transferred from peroxisomes or liposomes containing PI (4,5) P2 to the ER in vitro, and the presence of peroxisomes promotes the transfer of cholesterol from lysosomes to the ER [16]. The uptake and direct use of preformed fatty acids and cholesterol in the manner described above, rather than synthesizing them de novo*,* represent a significant energy-saving strategy for cells whose environment is often depleted of the relevant anabolic nutrients or which are being utilized for other tumor-sustaining purposes.

### Sources

Glucose, glutamine and acetate are the main source for de novo lipid synthesis, with the former being the most important dietary source. Acetate, derived directly from dietary sources, the gut microbiome, intracellular deacetylation reactions or directly from pyruvate, is converted into acetyl-CoA in the cytoplasm for lipid synthesis [17–19]. Glutamine can be utilized as an anaplerotic TCA cycle substrate and then, via reverse carboxylation, be converted to citrate, transported to the cytoplasm and then converted to acetyl-CoA via the action of ATP citrate lyase (ACLY) [17–19]. Ammonia, released from glutamine, activates the dissociation of glucose-regulated, N-glycosylated sterol regulatory element-binding proteins (SREBPs)-cleavage-activating protein (SCAP) from insulin-inducible gene protein (Insig), an endoplasmic reticulum-retention protein, leading to SREBP translocation and lipogenic gene expression to promote lipogenesis. SCAP is a critical sensor of glutamine, glucose and sterol levels to precisely control lipid synthesis [20].

### De novo* lipid synthesis*

Key regulatory factors and enzymes of lipogenesis include SREBPs, acetyl coenzyme A carboxylase (ACC), ACLY and fatty acid synthase (FASN), all of which are up-regulated to varying degrees in human cancers [21]. SREBPs are a family of basic helix–loop–helix leucine zipper transcription factors that regulate de novo fatty acid and cholesterol synthesis as well as cholesterol uptake [22]. Of the three subtypes of SREBPs, SREBP1a regulates fatty acid and cholesterol synthesis and cholesterol uptake, SREBP1c mainly controls fatty acid synthesis and SREBP-2 overlaps functionally with SREBP1 to regulate cholesterol synthesis and uptake [22]. EGFR signaling increases glucose uptake to promote N-glycosylation of SCAP. Glycosylation stabilizes SCAP and reduces its association with Insig-1, allowing movement of SCAP/SREBP to the Golgi and consequent proteolytic activation of SREBP, leading to increasing fatty acid synthesis and tumor growth [23]. ACLY is a downstream target of SREBPs and, via its conversion of cytoplasmic citrate to acetyl-CoA, serves to generate the both the most lipid precursor and the substrate for acetylation reactions [24]. Acetyl-CoA synthases (ACSSs) convert acetate into acetyl-CoA, thus ensuring a proper balance between these two pools of critical substrates. Following the conversion of citrate and acetate to acetyl-CoA, ACC catalyzes the ATP-dependent carboxylation of acetyl-CoA to malonyl-CoA, which is used in the synthesis of fatty acids. FASN is a key and rate-limiting lipogenic enzyme that catalyzes the last step in de novo fatty acid biogenesis [25].

### Lipid uptake, storage and secretion

CD36, also known as fatty acid translocase, transports fatty acids into cells and thus plays an important role in regulating the growth, metastasis and epithelial–mesenchymal transformation of many cancers [26]. Cholesterol can be synthesized de novo and also be obtained from intestinal absorption by internalizing it in the form of LDLs. LDLs bind to membrane-bound LDL receptors (LDLR), are internalized and then enter the lysosome where they release free cholesterol [27]. Sterol O-acyltransferase/acyl coenzyme A: cholesterol acyltransferase (SOAT1, SOAT2) attaches acyl-CoA to free cholesterol, generating CoA and cholesterol ester, and allowing the latter to be incorporated into lipid droplets [28]. ABCA1 is a plasma membrane transporter that promotes cholesterol export, thereby reducing intracellular levels [29, 30]. ABCA1 has particularly important roles in macrophages, where it promotes removal of excess cholesterol, thereby preventing their transformation into foam cells [31]. Transcription of ABCA1 is up-regulated by LXRs and RXR. In human macrophages, the LXRα-ABCA1 cholesterol efflux pathway is elevated by AMP-activated protein kinase (AMPK) [32].

### Fatty acid oxidation

Activation of peroxisome proliferator-activated receptor alpha (PPARα) induces the transcription of many genes related to mitochondrial beta type fatty acid oxidation (FAO) in the mitochondria and peroxisomes, thereby reducing lipid levels [33, 34]. Carnitine palmityl transferase I (CPT1) converts fatty acids to acyl carnitines, which are transported to the mitochondria where they are converted to fatty acyl-CoAs prior to entering the FAO cycle, thereby providing acetyl-CoA to drive the TCA cycle and produce ATP [35].

## Lipid metabolism reprogramming in tumors

**Fig. 1:**
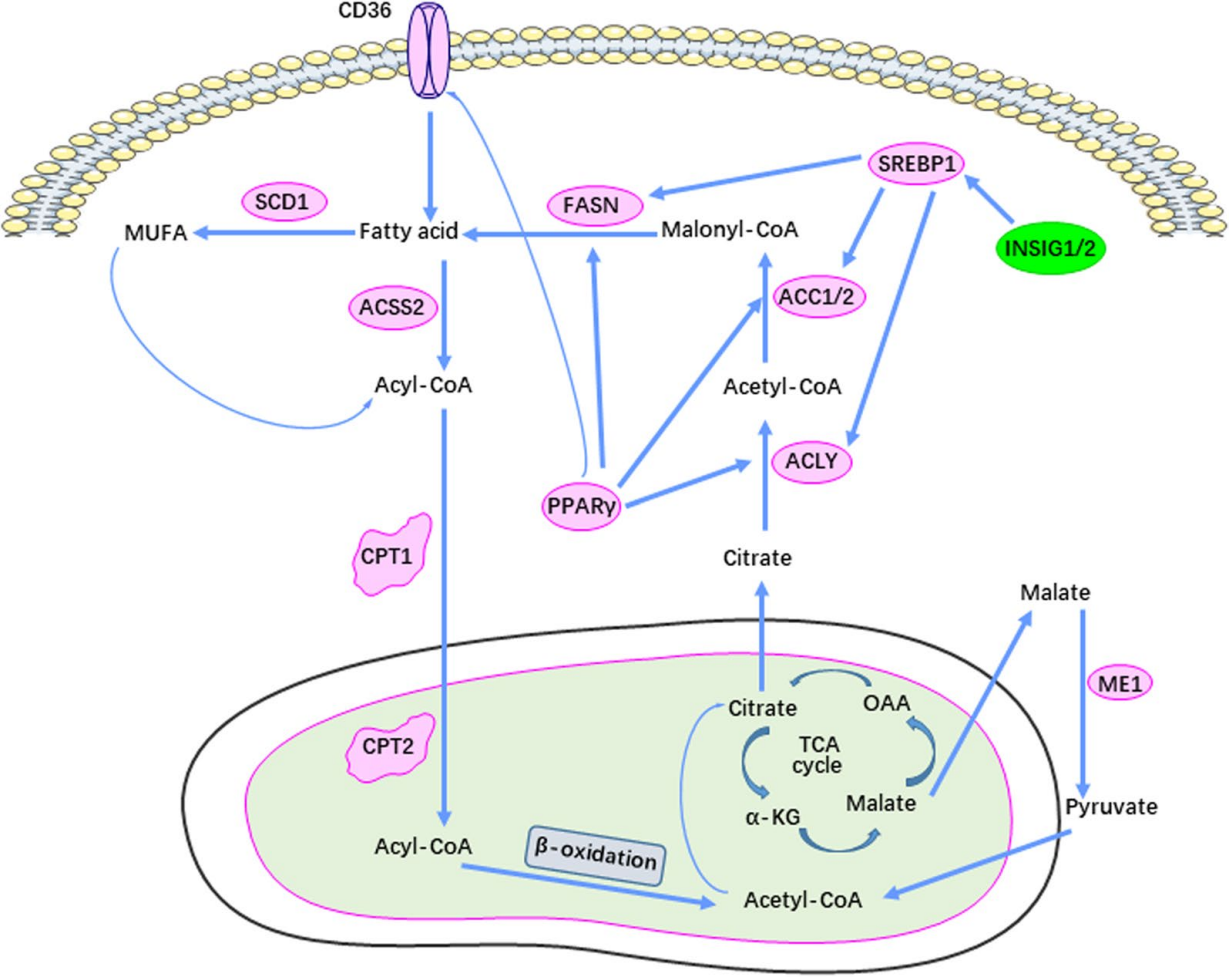
Signaling pathways of lipid metabolism in cancer. The magenta box indicates up-regulation of the respective enzyme and the green box indicates down-regulation

Lipid metabolism, specifically its synthesis, is significantly reprogrammed and up-regulated in cancer as is the uptake and storage of exogenous lipid (Fig. 1) [36–38]. It is generally believed that the metabolic characteristics of cancer cells are highly reliant upon lipid metabolism remodeling, including fatty acid regeneration and transport, lipid droplet formation and oxidation of these fatty acids to generate ATP [39].

Most tumors have abnormally active lipid metabolism, enabling them to synthesize, lengthen and desaturate fatty acids to support growth and rapid proliferation. Stearoyl-CoA desaturase (SCD)-dependent fatty acid desaturation is the most common way to desaturate fatty acids although some studies have shown that cancer cell lines may activate other desaturation pathways. Hepatocellular carcinoma (HCC) cells and primary hepatocytes desaturate palmitic acid to sapienate, a rare fatty acid that supports membrane biosynthesis during proliferation [40]. The utilization of other metabolites such as glucose and glutamine for fatty acid synthesis is a common feature of tumors. For example, glucose can be converted to fatty acids and cholesterol through de novo lipid biosynthesis pathways, metabolites of glucose and lipid are dynamically transported and then converted to other molecules in specific regions of cells [41].

Sphingolipid metabolism is generally up-regulated in tumors and inflammatory cells, with changes in sphingolipid balance being particularly prominent in colon cancer. Examples of sphingosine metabolism-related enzymes that are abnormally activated include sphingosine kinases (spHKs) 1 and 2. These produce sphingosin-1-phosphate (S1P), which promotes the development and progression of esophageal, gastric and colon cancers [42, 43]. S1P levels are elevated in both plasma and the peripheral lymphocytes of some cancer patients and S1P can initiate and/or support many inflammatory reactions, including lymphocyte infiltration of tumors [44]. In doing so, S1P is thought to promote the growth and progression of cancer cells, including proliferation, survival, migration, invasion and inflammation [45, 46].

## Lipid metabolism in the tumor microenvironment (TME)

While actual tumor cells have long been and continue to be the focus of many metabolic studies, those involving the tumor microenvironment (TME) and its non-transformed cellular populations are receiving increasing scrutiny [47]. Indeed, a full understanding of how and why tumors reorganize their metabolic landscapes requires an understanding of both cellular communities, along with an appreciation for how and why they communicate. Accumulation of the long-chain fatty acids in the TME enhances the differentiation of Th1 and Th17 cells and promotes dendritic cell maturation and T cell activation [48, 49]. Tumor-infiltrating CD8^+^ T cells accumulate large amounts of fatty acids from the TME via through CD36. This can induce lipid peroxidation and ferroptosis, leading to a loss of this tumor-inhibitory CD8^+^ population and allowing for unrestrained tumor growth. In support of this, inhibiting CD36 reduces lipid uptake by CD8^+^ cells, increases their survival and allows them to better suppress tumor growth [50].

Cholesterol and cholesterol esters also regulate CD8^+^ T cells proliferation and anti-tumor activity in many cancers [51]. OXPHOS and FAO can support Treg survival and differentiation, which is partially provided through fatty acids mediated by the AMPK and mTORC1 pathways [52]. Additional, M2-like macrophages and Treg cells also rely on FAO for both differentiation and activation during the course of tumorigenesis [53, 54]. Therefore, combined targeting of lipid metabolism and TME is a good strategy for tumor therapy [55].

### Characteristics of the TME

There are many differences between the TME and the normal tissue microenvironment, notably the former’s characteristically low oxygen tension, low pH and high pressure [56]. Tumor cells proliferate and migrate to distant locations because of their adaptive mechanisms to the low pH environment. Consequently, the TME’s acidity favors both local spread and metastatic dissemination [43, 56]. However, the acidic TME itself appears to play only a minor role in fatty acid uptake by tumor cells.

In addition to the poorly vascularized nature of the TME and a reduced ability to dispose of CO_2_, tumor cells contribute to TME acidity via several other cooperating and non-mutually-exclusive mechanisms. Among the most important of these is the tumor’s reliance on anaerobic metabolism (the Warburg effect), which produces large amounts of lactic acid. Another mechanism is the tumor cell’s reliance on a variety of membrane-localized ion exchange pumps, such as typical V-ATPases, that transport excess protons generated during metabolism to the outside of the cell in order to maintain the relative pH neutrality or alkalinity of the cytoplasm [57]. Finally, the low pH of the TME can form a positive feedback loop with some tumor cells, which promotes the secretion of TGF-β2. This stimulates PCK-Zeta-mediated CD36 translocation to promote fatty acid uptake [58], a much needed substrate under these conditions where both glucose and glutamine concentrations may be exceedingly low [59].

Fatty acids can be stored for extended periods of time in lipid droplets in the form of triglycerides through diacylglycerol O-acyltransferase 1 (DGAT1). This allows for their rapid mobilization and oxidation to generate ATP during times of energetic stress and/or when other nutrients are limiting [60]. Like the metabolic changes themselves, cancer cells’ interactions with the TME are complex and dynamic. Unsupervised clustering of gene expression changes involving fatty acid metabolism has shown that fatty acid biosynthesis pathways are often significantly up-regulated in tumor samples compared with adjacent normal samples [61].

While seemingly disorganized, a tumor is actually a complex, metabolically well-organized and unique tissue. Indeed, in addition to providing nutrients and maintaining the extratumoral environment, the TME also contains stromal cells, immune cells, vasculature and extracellular matrix (ECM) components, all of which are dependent on lipid metabolism [62, 63]. How these non-tumor cell types affect lipid disposition in tumor cells and how lipids in turn affect these components of the TME are of significant importance.

### Tumor-associated macrophages (TAMs)

TAMs regulate many processes associated with tumor progression, such as growth, drug resistance, metastasis, angiogenesis and immunosuppression. Tumors and the TME play important roles in polarizing macrophages, and intracellular lipid accumulation by these cells is key to this process [64]. In colorectal cancer, TAMs enhance lipid absorption through the CD36 cell surface receptor and derive energy in the form of ATP from the oxidation of fatty acids. Higher oxidative stress induced by fatty acid oxidation increases de-phosphorylation of STAT6-specific tyrosine phosphatase (SHP1) and Phos-tyr641-STAT6, which correlate with and presumably support the immunosuppressive and tumor-promoting functions of macrophages [65].

### Cancer-associated fibroblasts (CAFs)

CAFs are mainly derived from the tumor microenvironment [66]. Inhibition of arachidonic lipoxygenase 15 (ALOX15) by miR-522 in CAFs derived from gastric cancers reduced lipid-derived ROS and decreased chemotherapeutic sensitivity [67]. Heterogeneous nuclear ribonucleoprotein A1 (hnRNPA1) was found to mediate the accumulation of miR-522 into CAF exosomes, and ubiquitin-specific protease 7 (USP7) stabilized hnRNPA1 by de-ubiquitination [67].

### Mesenchymal stem cells (MSCs)

MSCs can affect drug resistance of gastric cancer cells in the TME. MSC co-culture improved drug resistance of gastric cancer (GC) cells. LncRNA histocompatibility leukocyte antigen complex P5 (HCP5) was induced in GC cells by MSC co-culture, contributing to drug resistance. Mechanistically, HCP5 sequestered miR-3619-5p and up-regulated PPARG coactivator 1 alpha (PPARGC1A), increasing transcription complex peroxisome proliferator-activated receptor (PPAR) coactivator 1α (PGC1α)/CEBPB and transcriptionally inducing carnitine CPT1, which prompted the fatty acid oxidation (FAO) in GC cells. These findings indicate that targeting HCP5 was a novel approach to enhancing the efficacy of chemotherapy in GC [68].

### Tumor-infiltrating dendritic cells (TIDCs)

Gene knockout or pharmacological inhibition of PPARα effectively removes immunologically dysfunctional TIDCs induced by fatty acid-carrying tumor-derived exosomes (TDEs), and characterized by increasing intracellular lipid content and mitochondrial respiration. Mechanically, TDE-derived fatty acids activate PPARα, which leads to excessive lipid drop biogenesis and enhanced FAO, metabolic transition to mitochondrial oxidative phosphorylation and dendritic cells immune dysfunction [69].

### T cells

T cells, which are effectors of anti-tumor immunity in certain solid tumors, compete with both tumor cells and non-malignant cellular components of the TME for limited nutrients. However, the TME in which lipids accumulate inhibits their ability to control tumor progression [70]. Pancreatic ductal adenocarcinoma (PDAC)-associated CD8^+^ T cells exhibit specific down-regulation of very long-chain acyl coenzyme A dehydrogenase (VLCAD), which leads to their accumulation of very long-chain fatty acids (VLCFAs) that mediate lipo-toxicity [71]. Tumor immunotherapy-activated CD8^+^ T cells mediate cell death mainly by inducing perforin–granzyme and the Fas–Fas ligand pathway [72].

Interferon-γ (IFN-γ) released from CD8^+^ T cells down-regulates the expression of SLC3A2 and SLC7A11, two subunits of the glutamate-cysteine antiporter system, thereby impairing the uptake of cysteine by tumor cells. IFN-γ also promotes lipid peroxidation and ferroptosis of tumor cells [73]. Effector memory CD4^+^ T cells respond differently to restricted supplies of glucose than other T cell subsets and maintain high levels of IFN-γ production in a malnourished environment [74].

Regulatory T cells (Treg cells) negatively regulate the immune system and play an important role in maintaining immune tolerance homeostasis. However, the accumulation of Tregs in the TME can hinder the anti-tumor immune response [75]. Generalized therapeutic targeting of Tregs leads to a systemic autoimmune responses and inflammation. Consequently, more specific and focused ways of specifically destroying Treg cells in tumors are needed for cancer immunotherapy. Enzymes and transcriptional factors of lipid metabolism including SREBPs, SCAP, FASN and FABP5 are potential targets in Treg cells whose inhibition might be associated with a more restricted anti-tumor response [76]. Gene enrichment analysis has shown lipid metabolism pathways to be enriched in Tregs from tumors compared to those from peripheral lymph nodes, with SREBP-target genes being particularly enriched. Tregs in tumor tissues maintain the functional status of TME-associated Tregs through the SREBP-dependent lipid synthesis pathway [77]. Specific inhibition of lipid synthesis and metabolic signaling of SREBPs in Tregs can release an effective anti-tumor immune response while sparing autoimmune toxicity [77, 78].

Immune checkpoint inhibitors (ICIs) have dramatically altered the prognosis of some advanced cancers, yet many patients still do not respond to treatment or relapse after relatively short responses or remissions [79]. If combined with ICIs, targeting tumor cell metabolism to regulate the immunosuppressive tumor microenvironment may achieve better effects. The loss of SCAP in tumor Tregs has been shown to increase the proportion of CD8^+^ T cells and Foxp3-CD4^+^ T cells in the TME, to inhibit tumor growth and to enhance anti-PD-1 immunotherapy. The proportion of Tregs and IFN-γ^+^ Tregs also decreased in tumor tissues but did not change in peripheral lymph nodes [80].

Fatty acid-binding proteins (FABPs) are a class of lipid chaperones that are required for intracellular lipid uptake and transport [81]. FABP5 inhibition in Tregs can cause mitochondrial DNA release and cGAS/STING-dependent type I IFN signaling, thereby increasing the production of regulatory cytokine IL-10 and promoting Treg inhibitory activity [82]. As a central metabolic regulator, CD36 is selectively up-regulated in intratumoral Tregs. CD36 fine-tunes mitochondrial fitness through the PPAR-β signaling pathway, enabling Tregs to adapt to the lactate-rich and acidic TME. CD36 silencing in Tregs inhibited tumor growth, decreased tumor-associated Tregs and enhanced the anti-tumor activity of tumor-infiltrating lymphocytes without significantly impacting immune homeostasis [83]. Targeting sphingosine kinase 1 (SK1) significantly enhanced the ICI response in mouse models of melanoma, breast cancer and colon cancer. Mechanistically, SK1 silencing was shown to reduce the expression of various immunosuppressive factors in the TME, thereby limiting the infiltration of Tregs [84].

T cells shape the immune responses in cancer, autoimmune diseases and infection via CD4^+^ T helper (Th) and CD8^+^ T cells. These responses are in turn suppressed by Treg cells [85]. Tregs inhibit the secretion of IFN-γ by CD8^+^ T cells, which can block the immunosuppressive (M2-like) TAMs-mediated activation of fatty acid synthesis [86]. Dysregulation of invariant natural killer T (INKT) cells in the tumor microenvironment hinders their anti-tumor effects. High levels of lactate in the TME reduced the anti-tumor immune response of INKT cells in the tumor [87]. PPARγ expression was inhibited in INKT cells, thereby reducing cholesterol (the substrate of IFN-γ) synthesis and IFN-γ production. After pharmacological activation of INKT cells, lipid biosynthesis was increased, and PPARγ and PLZF were synergistically promoted by enhancing the transcription of SREBF1. Hence, to promote lipid biosynthesis of INKT cells enhances the anti-tumor efficacy of immunotherapy [88].

### Tumor-associated myeloid cells (TAMs)

Although TAMS maintain an immunosuppressive microenvironment within tumors [89], identifying the myeloid specific receptors that modulate this function and that myeloid-derived suppressor cells (MDSC) remains a challenge. Members of the leukocyte immunoglobulin-like receptor B (LILRB) family are negative regulators of myeloid cell activation. LILRB2 antagonizes SHP1/2 receptor-mediated activation and enhances pro-inflammatory responses. In the presence of M-CSF and IL-4, LILRB2 antagonism inhibits the activation of Akt and STAT6 [90]. LIlRB2 blockers effectively inhibited granulocyte MDSC and Treg infiltration and significantly promoted the anti-tumor effect of T cell immune checkpoint inhibitors in vivo [91].

Polynucleate granulocytic MDSCs (PMN-MDSCs) are pathologically activated neutrophils that affect the anti-tumor immune response, thereby altering the efficacy of some tumor therapies. Fatty acid transporter 2 (FATP2) expression is specifically up-regulated in mouse and human PMN-MDSCs and plays a role by regulating arachidonic acid accumulation and the downstream synthesis of prostaglandin E2 (PGE2). Inhibition of FATP2 has recently been shown to reduce the growth of multiple tumor types, including lymphoma and lung, colorectal and pancreatic carcinomas [92].

Single-target therapies rarely take into account the immense molecular, biochemical and metabolic heterogeneity of most tumors, let alone that of the TME. Moreover, the TME often hinders the sensitization of effector lymphocytes, which reduces their infiltrative ability along with that of other effector cells, leading to impaired anti-tumor activity and the generation of so-called immunologically “cold tumors” [93]. More global multi-target drug design should thus consider not only the tumor cells themselves, but also the TME and its contents so as to achieve better therapeutic effects and fewer toxicities. The challenge is further complicated by the marked differences in intratumoral TME contents of regions within close geographical proximity of one another and by the rapidity with which the TME environment can adapt to environmental alterations [94, 95].

## Posttranslational modifications (PTMs) in cancer lipid metabolism

PTMs at specific amino acid residues of proteins are necessary for and mediate almost all dynamic processes within cellular signaling networks [96–100]. Protein PTMs include, but are not limited to, phosphorylation, acetylation, ubiquitination, SUMOylation, succinylation and methylation [99–104]. These alterations, which are often transient in nature, affect the structure, function, stability and localization of many proteins [96, 100, 105, 106]. PTMs may be differ considerably between tumors and their normal tissue counterparts and often serve as driving forces for tumorigenesis [107–110].

### Phosphorylation and de-phosphorylation in cancer lipid metabolism

**Fig. 2:**
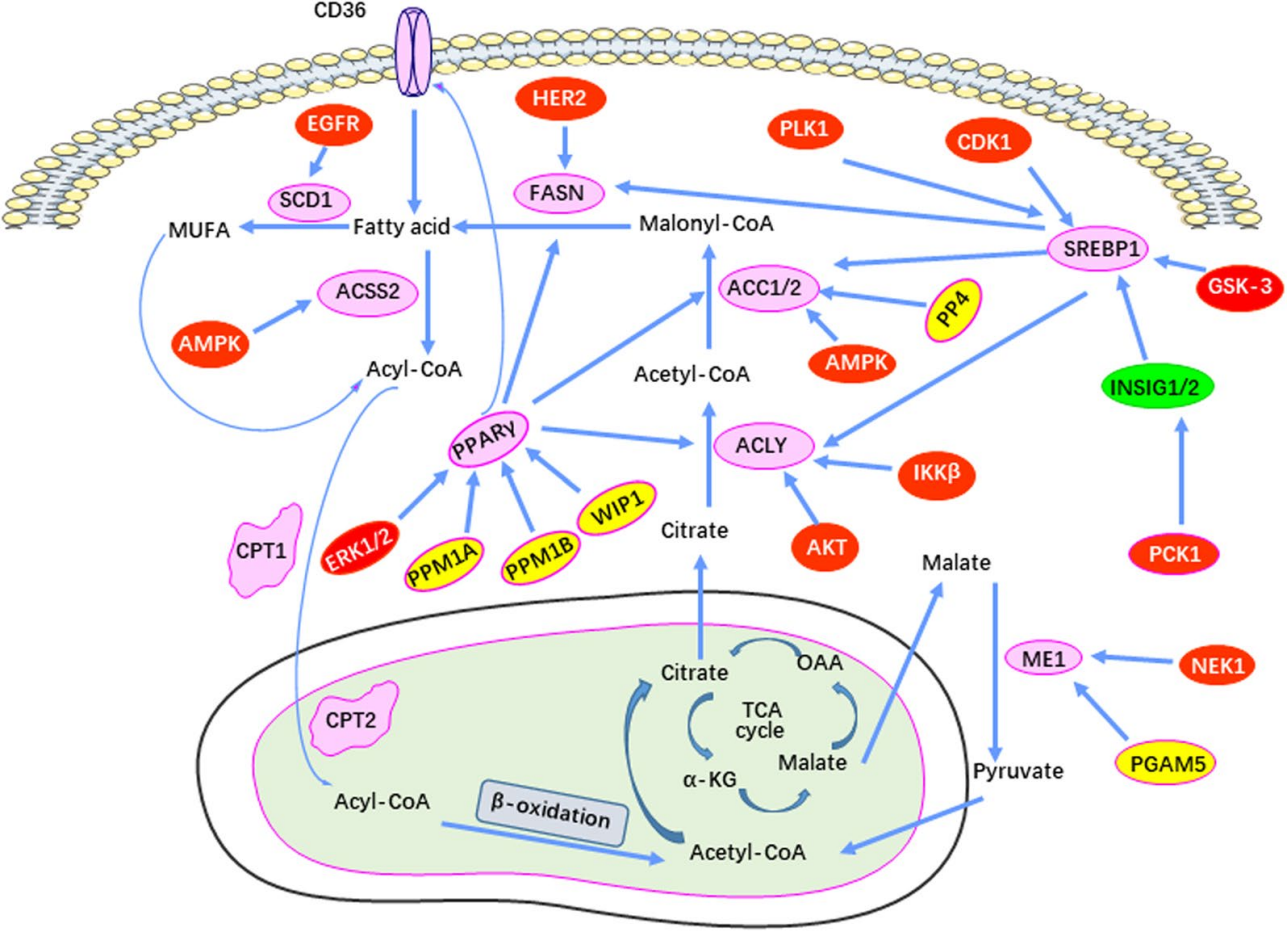
The phosphorylation-mediated control of lipid metabolism enzymes during tumorigenesis. The red boxes represent protein kinases, which mediate the phosphorylation of these lipid-metabolizing enzymes, and the yellow boxes represent phosphatases, which are responsible for de-phosphorylation of lipid metabolism enzymes during tumorigenesis

Phosphorylation is among the most widely studied PTMs involved in lipid metabolism reprogramming in tumors (Fig. 2). In response to aberrant oncogenic signaling, protein kinases may either activate or inhibit the activities of their target proteins while also affecting subcellular localization and the interaction with other proteins in ways that are not necessarily mutually exclusive. Collectively, these various PTMs serve to regulate cell growth, differentiation, apoptosis, metabolism and cell signal transduction in both normal or transformed cells [111–113]. The most frequently phosphorylated amino acids are threonine, serine and tyrosine [114]. Phosphatases, which reverse the PTMs mediated by their attendant kinases, normally function in tandem to maintain the proper balance between the phosphorylated and un-phosphorylated state. This balance is often altered by the aberrant signaling pathways that are activated in many tumors [115–119].

Protein kinases are one of the largest gene families [120]. Although tyrosine kinases are the most important class of kinases that regulate cell growth and differentiation, the serine/threonine kinases are actually more numerous [109]. Altered phosphorylation patterns can lead to serious consequences, such as the development of cancer. Indeed, the first identified function of an oncogene was the constitutively active tyrosine kinase activity of the transforming avian retroviral protein v-src [121]. Therefore, drugs targeting phosphorylation pathways, particularly kinases themselves, have long been a promising area for cancer therapy [122].

The AMPK complex senses intracellular ATP levels and plays an important role in maintaining cellular energy supplies. Under low-energy conditions, AMPK phosphorylates specific enzymes and growth control nodes, thereby increasing ATP production and reducing ATP consumption. As might be expected, functions that are stimulated by AMPK activation include the major energy-generating pathways of glycolysis, OXPHOS and FAO, whereas energy-consuming functions such as those centered around protein translation and proliferation are inhibited. Collectively, the re-wiring of these pathways allows the energy-depleted cell to regain a normal energy balance and which point AMPK signaling is mitigated [123, 124].

AMPK depletes liver lipid content by reducing the activity of mTORC and inhibiting the expression of SREBP1c. In mice, AMPK also phosphorylates SREBP1c at Ser372, decreased SREBP1c nuclear localization and inhibition of the diet-induced hepatic steatosis of insulin resistance [125]. mTORC is an important medium for regulating cell metabolism and growth and promoting SREBP-dependent fatty acid synthesis [126, 127].

ACC is phosphorylated and inactivated by AMPK and many other kinases [128]. AMPK phosphorylates functionally homologous sites on ACC1 and ACC2 (Ser79 and Ser219, respectively) thereby inhibiting their activities. Phospho-ACC1 Ser79 thus serves as a general indicator of AMPK activity [129, 130]. TGF-activated kinase 1 (TAK1) mediates ACC1 inhibitory phosphorylation, which promotes an increase in cell acetylated coenzyme A, thereby promoting the acetylation and activation of Smad2 transcription factor and ultimately inducing epithelial–mesenchymal transformation and metastasis [131].

Phosphorylation and de-phosphorylation play important roles in de novo fatty acid biosynthesis. Mice maintained on high-fat diets contain high levels of USP30, which de-ubiquitinates and stabilizes ACLY and FASN. IKK-beta directly phosphorylates ACLY and promotes its interaction with USP30, thereby increasing USP30-mediated de-ubiquitination of ACLY and fatty acid biosynthesis [132].

Phosphorylation and de-phosphorylation reactions are also important for cholesterol synthesis. In the liver, the rate-limiting enzyme of the pathway, hydroxymethyl glutaryl coenzyme A reductase (HMGCR), has been shown to be significantly up-regulated after feeding via a mechanism involving its interaction with USP20. Elevated postprandial glucose and insulin levels stimulate mTORC1 to phosphorylate S132 and S134 in USP20. This licenses USP20’s recruitment into the HMGCR complex and antagonizes its degradation, thereby stabilizing HMGCR and promoting cholesterol synthesis [133]. In contrast to this positive control, the phosphorylation of HMGCR at Ser872 can inhibit AMPK-mediated activation of HMGCR, which may be a potential mechanism of hypercholesterolemia and related cancers [134].

Phosphorylation/de-phosphorylation also plays an important role in FAO. In colon cancer cells, PKC Zeta interacts with SIRT6 following their exposure to palmitic acid. PKC Zeta phosphorylate T294 of SIRT6, which facilitates SIRT6’s interaction with chromatin. T294 phosphorylation is required for SIRT6’s localization to and deacetylation of chromatin, particularly around the promoters of genes such as acyl-CoA synthetase long-chain family member 1 (ACSL1), CPT1, carnitine–acylcarnitine translocase (CACT) and HADHB and then induces the expression of these genes to mediate FAO [135]. In conclusion, the balance between phosphorylation and de-phosphorylation is normally responsible for maintaining proper levels of lipid metabolism. Reprogramming of several of these pathways is important for maintaining the growth of certain tumors and represents potential points of therapeutic intervention.

### Acetylation and deacetylation in cancer lipid metabolism

Acetylation is a key posttranslational modification that coordinates metabolic flow with circadian rhythms, cell cycle and energy production. Lysine acetyltransferases (KATS) and lysine deacetylases (KDACs) are responsible for regulating reversible protein acetylation [136].

**Fig. 3:**
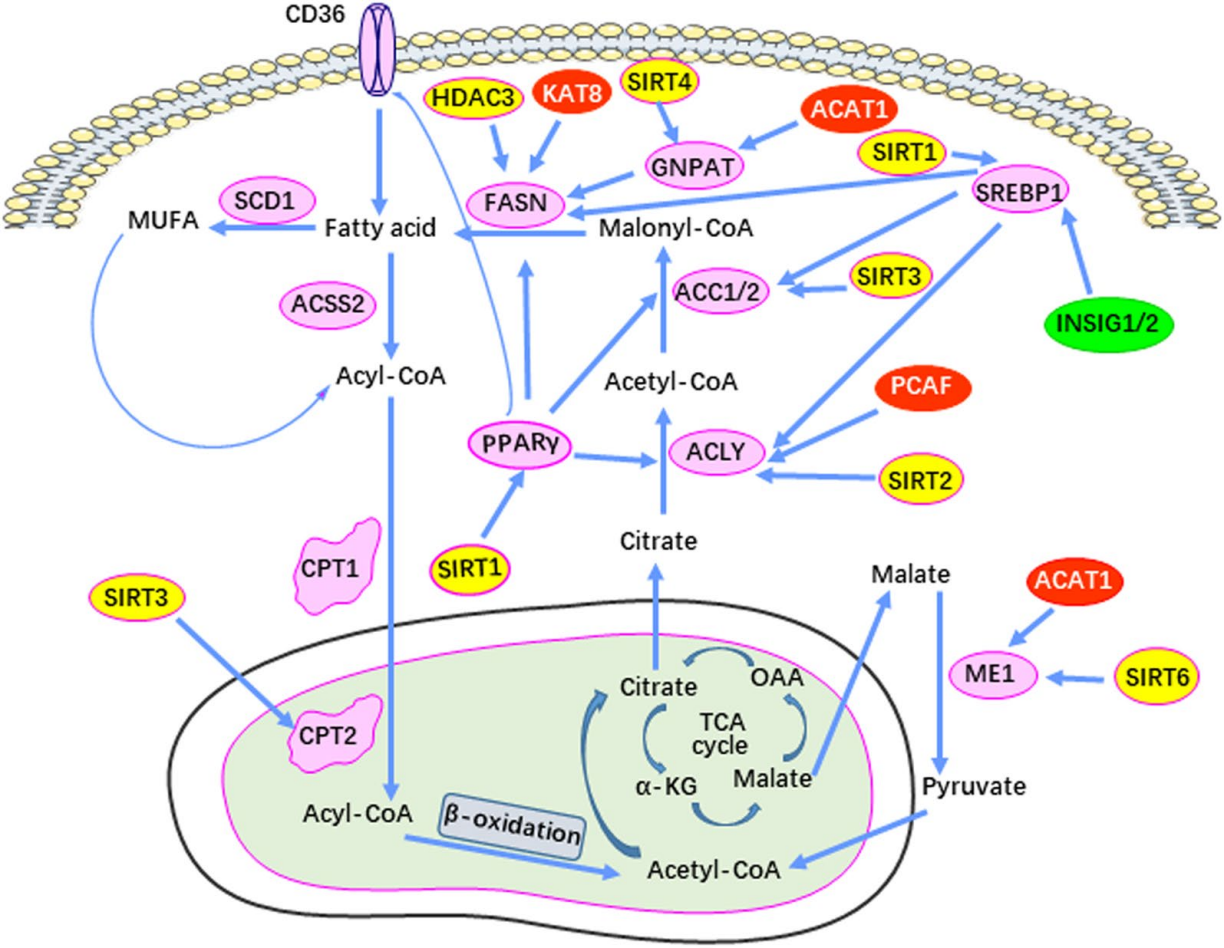
Acetylation-mediated control of lipid metabolism enzymes during tumorigenesis. Red boxes represent acetylation transferase, which mediates the acetylation of lipid metabolism enzymes. Yellow boxes indicate deacetylases, which remove the acetylation modification of lipid metabolism enzymes during cancer development

Acetylation and deacetylation affect de novo lipid synthesis (Fig. 3). For example, lipin 1 is a phospholipid acid phosphorylase that plays an important role in lipid metabolism. In growth factor-deficient mammalian cells, GSK-3 kinase activates the acetyltransferase TIP60 (TIP60-Ser86) and catalyzes the acetylation of ULK1 (Atg1), thereby activating autophagy [137]. TIP60’s direct acetylation of lipin 1 promotes the latter’s translocation to the endoplasmic reticulum, thereby promoting triglyceride synthesis. SIRT1 deacetylates lipin 1 and inhibits the synthesis of triglycerides [138]. ACAT1-mediated acetylation of dihydroxyacetone phosphate acyltransferase (GNPAT) plays a key role in FASN stabilization to increase lipid synthesis in hepatocellular carcinoma. ACAT1 is up-regulated by excess palmitic acid and acetylates GNPAT K128, which in turn inhibits TRIM21-mediated ubiquitination and degradation of GNPAT. In contrast, SIRT4 antagonizes the function of ACAT1 by antagonizing deacetylation of GNPAT. Acetylated GNPAT competes with FASN to bind TRIM21, which inhibits TRIM21-mediated FASN degradation to enhance lipid synthesis [139]. DHA (Docosahexaenoic acid), an omega-3 polyunsaturated fatty acid, down-regulates SREBP1. DHA induces SIRT1 expression in CCD841CON human colon epithelial cells. SIRT1 deacetylates SREBP1 to inhibit intracellular signal transduction mediated by SREBP1, including downstream lipid synthesis pathways and COX_2_-involved angiogenesis [140].

Acetylation and deacetylation can affect FAO. Low levels of palmitate activate the CDK1-SITR3-CPT2 cascade in liver. SIRT3 catalyzes the deacetylation and dimerization of CPT2, promoting mitochondrial FAO and protecting the liver from lipid toxicity [141]. In hepatic stellate cells (HSC), SIRT1 can deacetylate PPAR-γ to prevent activation of hepatic stellate cells (HSC), thus protecting the liver from fibrosis and sclerosis [142].

### Ubiquitylation and de-ubiquitination in cancer lipid metabolism

Ubiquitylation is an important PTM that plays a crucial role in regulating the levels and activities of numerous metabolic enzymes and ensures proper control over intracellular homeostasis. The regulation of ubiquitination itself is multifaceted, not only at the transcriptional and posttranslational levels (phosphorylation, acetylation, methylation, etc.), but also at the translational level [143].

**Fig. 4:**
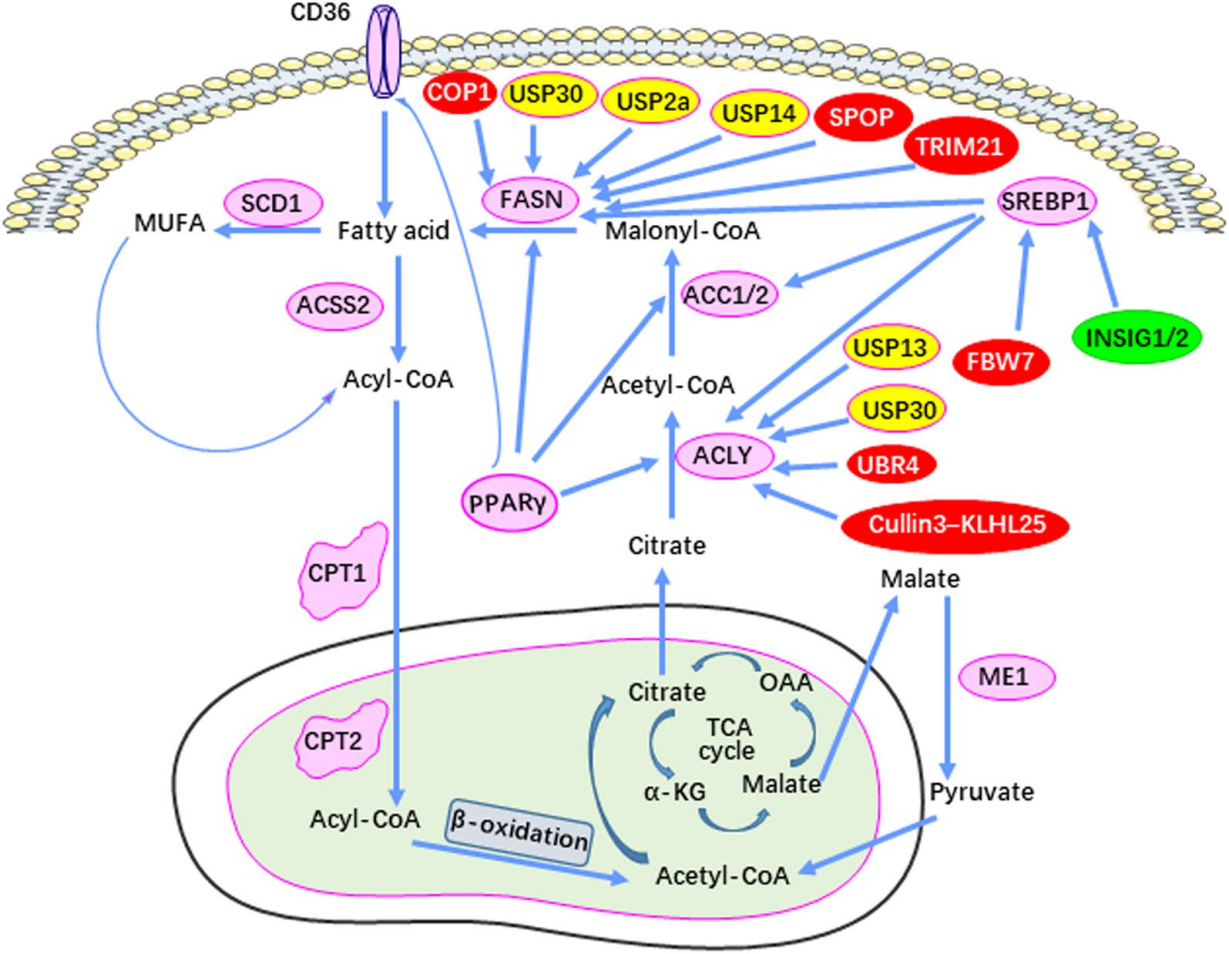
Ubiquitination-mediated control of lipid metabolism enzymes in tumorigenesis. The red box represents ubiquitinases, which regulate the ubiquitination of lipid metabolism enzymes. Yellow boxes indicate de-ubiquitinating enzymes

Ubiquitylation is an ATP-dependent cascade that links ubiquitin oligomers of variable length to proteins. Ubiquitin (Ub) is a highly conserved regulatory protein containing 76 amino acids whose covalent attachment to its targets occurs via a cascade of step-wise enzymatic reactions that mediate Ub activation (E1), Ub binding (E2) and Ub linking (E3). Ub’s sites of attachment include seven of its lysine residues (K6, K11, K27, K29, K33, K48 and K63). Different Ub chain lengths and different sites of attachment (both on Ub and its targets) lead to different fates of its substrates. K48 polyubiquitination is one of the most widely studied types and is primarily used to label proteins that are recognized and degraded by the 26S proteasome [132, 133]. The E3 reaction involves a large number of Ub ligases whose sites of targeting on their protein substrates are associated with different outcomes. Collectively, the lysine substrate attachment sites on Ub, the site(s) of its attachment on its substrates and the number of Ub molecules at these sites exert highly sensitive and specific control over the fate and function of the modified substrate. Abnormalities in each of these regulatory process may lead to serious human diseases, including cancer (Fig. 4) [144].

In contrast to the ubiquitinating enzymes, those involved in de-ubiquitination (DUBs) catalyze the removal of Ub and are relatively few in number. However, they also exhibit targeted specificity and have a decisive effect on cellular functions such as cell cycle progression, apoptosis, receptor regulation and gene transcription [145, 146].

Ubiquitylation/de-ubiquitination also affects lipid synthesis. For example, the de-ubiquitination enzyme USP30 is highly expressed in HCC. IKK-beta phosphorylates USP30 S210/S364. This stabilizes USP30, which in turn de-ubiquitinates and stabilizes ACLY and FASN to increase lipid synthesis [132]. As an adaptor, TRB3 binds the E3 ubiquitin ligase COP1 to ACC1/2 and thereby mediates the proteolysis of ACC1/2 in a UB-dependent manner while inhibiting fatty acid synthesis and stimulating lipolysis [147]. COP1 also ubiquitinates FASN and uses as an adapter protein SH2-tyrosine phosphatase (SHP2) whose SH2 domain is critical for the interaction [148]. Activated Akt inhibits FASN ubiquitination through the de-ubiquitination enzyme USP2A, thereby promoting lipid synthesis in HCC [149].

Ubiquitylation and de-ubiquitination also affect cholesterol metabolism. High sterol concentrations can induce HMGCR degradation. Ring finger protein 145 (RNF145) is a UB ligase that interacts with the ER protein Insig-1/2. RNF145 and gp78, each acting as sterol responsive ER-resident E3 ligases, independently coordinate the ubiquitination-mediated degradation of HMGCR. The UBE2G2-dependent E3 ligase HRD1 partially regulates the stability of HMGCR. UBXD, also an E3 ligase, can mediate steroid-induced ubiquitination and degradation of HMGCR [150–152].

Cholesterol synthesis is very sensitive to oxygen levels. In response to hypoxia, hypoxia-inducible factor 1 alpha (HIF-1α) activates INSIG-2 transcription, leading to the accumulation of INSIG-2 protein, which binds to HMGCR and accelerates its ubiquitination and degradation, thus contributing to proliferative inhibition [153]. Abnormal cholesterol metabolism in multidrug-resistant cancer cells leads to decreased E3 ligase Trc8, up-regulation of HMGCR and enhanced cholesterol synthesis [154].

Squalene mono-oxygenase is an important control point in the pathway of cholesterol synthesis and is regulated at the posttranslational level by accelerated cholesterol-dependent ubiquitination, thereby leading to proteasomal degradation and squalene accumulation [155]. The E3 UB ligase DOA10/TEB4 promotes the degradation of squalene mono-oxygenase [156]. LXR transcriptionally induces the IDOL E3 Ub ligase, and IDOL ubiquitinates the cytoplasmic domain of the low-density lipoprotein receptor (LDLR), thus targeting its degradation and inhibiting the LDL uptake [157].

### SUMOylation and de-SUMOylation in cancer lipid metabolism

Many studies have shown that ubiquitination/de-ubiquitination imbalance is an important cause of tumorigenesis. Small ubiquitin-like modifier (SUMO) is similar to ubiquitin in its three-dimensional structure, despite differing in its amino acid sequence and surface charge distribution. There are four types of SUMO subtypes: SUMO1-4, with SUMO2 and 3 being the most closely related. With the exception of SUMO4, they are widely distributed in human tissues [158].

Unlike ubiquitination, which tends to promote proteolysis, SUMOylation more commonly reduces the degradation of modified proteins by regulating protein–protein interactions as well as subcellular localization and function. By analogy to ubiquitination reactions, the reversible binding of SUMO molecules to their substrates is catalyzed by a cascade of enzymes (E1 activators, E2-binding enzymes, E3 ligases and SENPs) that mediate maturation, activation, conjugation, ligation and de-SUMOylation [159].

SUMOylation and de-SUMOylation significantly impact the transcriptional regulation of lipid metabolism. In HCC, up-regulation of SUMO1 induces ubiquitin-like modification of K751 of large tumor suppressor (LATS1), resulting in instability of its phospho-T1079 site and attenuation of LATS1 kinase activity. SUMO1 up-regulation also SUMOylates CPAP K921 and K975, which are necessary for CPAP to act as a coactivator of NF-κB [160, 161]. SUMO2 up-regulation catalyzes ubiquitination of liver kinase B1 (LKB1) K178 and impedes nuclear-to-cytoplasmic transport [162].

SUMO-specific protease I (SENP1) enhances HIF-1α stability and transcriptional activity in hypoxic HCC cells by de-SUMOylating the K391 or K477 residues of HIF-1α. On the other hand, without affecting HIF-1α protein levels, CBX4 promotes HIF-1α-K391/K477 SUMOylation and enhances HIF-1α transcriptional activity to promote lipid metabolism and angiogenesis in tumors [163, 164]. SUMO1 up-regulation also induces a variety of other effects, including the disintegration of TBL1-TBLR1 from the NCOR complex and increased transcriptional activity [165], as well as increased nuclear transport of KLF5 [166].

### Methylation and de-methylation in cancer lipid metabolism

Arginine methylation affects many biological processes in mammalian cells, including transcription, metabolism, signal transduction, mRNA translation, receptor transport and protein stability [167]. The protein arginine methyltransferases (PRMTs) are a family of nine enzymes that regulate the stability, cellular localization and activity of substrates that include histones, transcription factors and other proteins [168]. PRMTs transfer one or two methyl groups from S-adenosylmethionine (SAM) to the guanidine nitrogen atom of arginine to form methylarginine and the metabolite S-adenosylhomocysteine (SAH) [169].

Methylation/de-methylation reactions regulate lipid metabolism in many tumor types. For example, PRMT5-mediated symmetric di-methylation of SREBP1a at R321 is associated with HCC progression and poor prognosis. This methylation excludes the phosphorylation of S430 of SREBP1a by GSK-3β resulting in the dissociation of SREBP1a from FBXW7 and its inability to be ubiquitinylated and proteasomally degraded. In this manner, the now stabilized SREBP1a can participate in de novo lipid synthesis, thus contributing to tumor growth and proliferation [170].

## Cross talk between different posttranslational modifications in cancer lipid metabolism

**Fig. 5:**
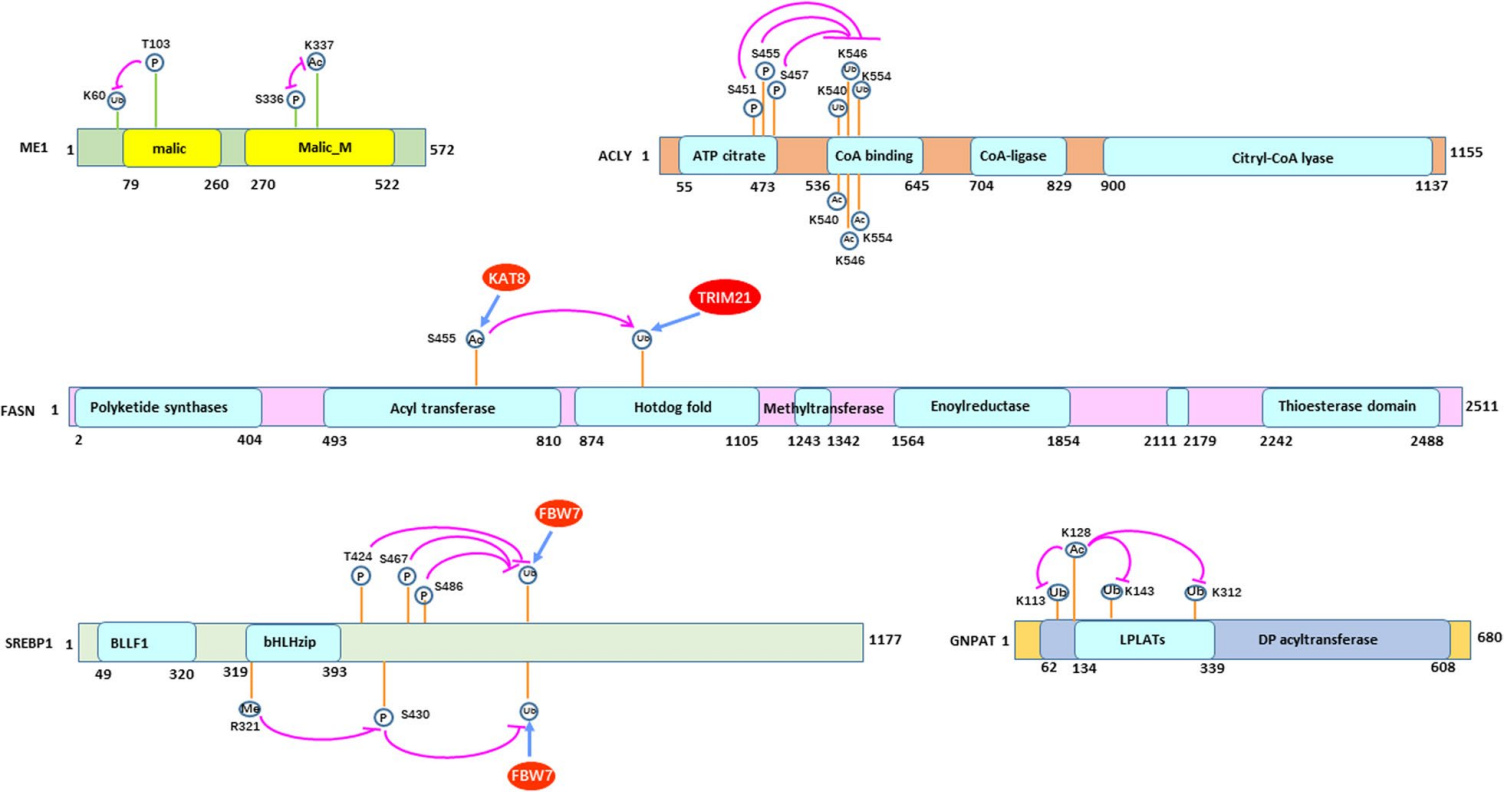
Cross talk between different PTMs in cancer lipid metabolism. Domains are drawn to scale. S, Serine; K, lysine; P, phosphorylation; Ub, ubiquitination; Ac, acetylation; ME1, malic enzyme 1; ACLY, ATP citrate lyase; SREBP1, sterol regulatory element-binding protein 1; GNPAT, glyceronephosphate O-acyltransferase; LPLATs, lysophospholipid acyltransferases; DP acyltransferase, dihydroxyacetone phosphate acyltransferase; BLLF1, Herpes virus major outer envelope glycoprotein; and bHLHzip, basic helix–loop–helix–zipper domain

Although cross talk among different PTMs is common, it has been poorly studied. The interactions among PTMs are complex and likely reflect variations in charge, steric hindrance, the resulting conformational changes mediated by each PTM and the different environments in which they reside. Interactions between the known PTMs that affect cancer lipid metabolism-related enzymes are summarized in Fig. 5.

Malic enzyme 1 (ME1) generates NADPH for fatty acid biosynthesis via the reversible oxidative decarboxylation of malate and the production of pyruvate. S336 phosphorylation and K337 acetylation are mutually exclusive PTMs at adjacent sites in ME1. NEK1 kinase-mediated phosphorylation of S336 antagonizes acetylation at K337. In contrast, de-phosphorylation of this site by the PGAM phosphatase increases ACAT1-mediated acetylation of K337. This event licenses ME1 dimerization and activation, thus enhancing NADPH generation, lipogenesis and the promotion of colorectal tumorigenesis. In contrast, SIRT6-mediated deacetylation of K337 antagonizes ACAT1 by restoring inhibitory S336 phosphorylation and inhibiting ME1 homo-dimerization [171].

Competition between acetylation and ubiquitination for the same lysine residues in ACLY regulates fatty acid synthesis and cell growth responses to glucose [172]. In HCC, and in the presence of glucose, the P300-related factor acetyltransferase acetylates the 540, 546 and 554 (3 K) lysine residues of ACLY by blocking ubiquitination and degradation thereby stabilizing the protein and promoting lipid biosynthesis. In contrast, Sirtuin2 deacetylates and de-stabilizes ACLY by allowing for ubiquitination of the same sites and proteasome-mediated degradation. Importantly, 3 K acetylation of ACLY is increased in human lung cancer [173].

In HCC, IKK-β can phosphorylate S451, S455 and S457 of ACLY, which mediates an association between ACLY and USP30 and promotes the latter’s de-ubiquitination at K540, K546 and K554, its stabilization and a resulting increase in fatty acid synthesis [132]. Also in HCC, ACAT1 mediates GNPAT acetylation at K128, which inhibits TRIM21-mediated ubiquitination of GNPAT at K113, K146 and K312 thereby stabilizing GNPAT by excluding the ubiquitylation of these resides and slowing proteasomal degradation [139].

PRMT5 binds SREBP1a and catalyzes its symmetric di-methylation of R321. This prevents the GSK-3β mediated phosphorylation of SREBP1a S430, resulting in the dissociation of SREBP1a from the F-Box protein FBXW7 and the failure to degrade SREBP1a via the proteasome [170].

The Polo-like kinase Plk1 mediates the phosphorylation of T424, S467 and S486 of nuclear SREBP1, which abrogates its degradation by disrupting its association with FBXW7 in cervical cancer [174]. There FBXW7 also interacts with nuclear SREBP1a and enhances its ubiquitination and degradation in a manner that is also dependent on the GSK-3β-mediated phosphorylation of T426 and S430 [175]. Finally, in colorectal cancer, KAT8-mediated acetylation of FASN promotes its association with the E3 ubiquitin ligase TRIM21, which enhances its proteasome-mediated degradation [176].

## Targeting reprogramed lipid metabolism and PTMs of cancer lipid metabolism enzymes for cancer treatment

A variety of approaches have been proposed for capitalizing on the differences in lipid metabolism between normal and malignant cells for the purpose of achieving therapeutic benefit. Many inhibitors of fatty acid metabolic enzymes have been designed toward this goal. Targeting FASN effective inhibits the growth of most cancers including liver, colorectal and pancreatic cancers, largely by inducing tumor cell death [177]. FASN inhibitors that repress tumor growth both in vitro and in mouse models include cerulenin, orlistat, C57, C93, IPI-9119 and TVB-2640 [178, 179]. We recently showed that combining the ACLY inhibitor ETC1002 and anti-PD-L1 antibody therapy significantly inhibited the incidence and growth of DEN-induced HCC [132]. Similarly, the SOAT1 inhibitor avasimibe has effectively inhibited the growth of high-cholesterol/high-fat diet (HCHFD)-induced HCC and colorectal cancer by inhibiting cholesterol esterification [180, 181]. Both the HMGCR inhibitor simvastatin and the squalene epoxidase inhibitor terbinafine repress the induction of HCC by blocking de novo cholesterol synthesis [182, 183]. The ACC1 inhibitors soraphen A, 5-tetradecyl-oxy-2-furoic acid and ND-646 block the growth of many cancer cell types by inhibiting de novo fatty acid synthesis and enhancing FAO [184, 185]. Sulfo-N-succinimidyl oleate, a CD36 inhibitor, reduced cancer cell migration and growth by repressing fatty acid uptake [186]. The SREBPs inhibitors fatostatin and betulin decreased invasion and progression of many cancer types by reducing lipogenesis [187, 188]. Active ongoing or completed clinical trials on targeted agents for the cancer lipogenesis enzymes/pathways are summarized (Table 1). These studies suggest that targeting fatty acid metabolism showed potential anti-tumor effects in many cancer types.

**Table 1:** Active ongoing or completed clinical trials on targeted agents for the cancer lipogenesis enzymes/pathways

Drugs	Targeted protein	Cancer type	Function	ClinicalTrial. gov identifier	Status
TVB-2640	FASN	Advanced tumors	To inhibit FASN	NCT02223247	Completed
TVB-2640	FASN	Breast and colon cancer	To inhibit FASN	NCT03179904	Recruiting
CLA	FASN	Breast cancer	To inhibit FASN	NCT00908791	Completed
Statins	HMGCR	Solid tumors	To inhibit HMGCR	NCT02285738	Completed
Metformin	AMPK	Acute lymphoblastic leukemia	To activate AMPK	NCT03118128	Recruiting
Metformin	AMPK	Breast, endometrial, Prostate cancer	To activate AMPK	NCT01620593	Completed
Lapatinib	AMPK	Metastatic breast cancer	To activate AMPK	NCT01477060	Terminated
RGX-104	LXRs	Lung and endometrial cancer	To activate LXRs	NCT02922764	Recruiting
NRX194204	RXR	Non-small cell lung cancer	agonist of RXR	NCT01540071	Active
Bexarotene	RXR	Cutaneous T-cell lymphoma	To activate RXR	NCT01007448	recruiting
Pioglitazone	PPARa	Bladder cancer	agonist of PPARa	NCT01637935	Completed
TPST-1120	PPARa	Hepatocellular carcinoma	antagonist of PPARα	NCT03829436	Active

To this end, targeting the enzymes responsible for the PTMs of cancer lipid metabolism enzymes has thus rightfully attracted much attention (Table 2). In mammals, cholesterol biosynthesis increases after feeding but is inhibited during fasting. In response to feeding, USP20 stabilizes HMGCR. The small molecule GSK2643943A targets USP20 and de-stabilizes HMGCR, without affecting the total of phosphorylated levels of USP20. As a result, GSK2643943A significantly decreases diet-induced weight gain and serum and liver lipid levels while improving insulin sensitivity and increasing energy consumption [133]. Non-alcoholic steatohepatitis (NASH) and cirrhosis are predisposing factors for HCC development [189]. Thus, the prevention or reversal of hepatic inflammation and the ensuing hepatic fibrosis should reduce the incidence of this deadly neoplasm. The FXR-Plin1 cascade could thus be an important target for drug discovery and treatment in liver fibrosis. However, activated HSCs show only a limited response to some FXR agonists due to enhanced FXR SUMOylation, possibly due to enhanced FXR SUMOylation and decreased FXR protein levels in the process of liver fibrosis. SUMOylation of FXR in HSCs is mainly mediated by SUMO1 at K122, K275 and E277, which could be a potential target for inhibition of FXR degradation. In various models of NASH and hepatic fibrosis, the combination of SUMOylation inhibitors such as SP (a natural product aminocyclitol produced by *Streptomyces spectabilis*) and GA (anacardic acid isolated from the seed coat of *Ginkgo biloba*) together with OCA (obeticholic acid, a potent FXR agonist) inhibited HSC activation and fibrogenesis. Such combination of FXR agonists and SUMOylation inhibitors represents potentially promising strategies for reversing or preventing hepatic fibrosis in response to a variety of etiologies, including toxins, cholestasis and NASH [190–192]. Ulixertinib reduced ME1 T103 phosphorylation and promoted ME1 protein degradation, which dramatically inhibited spontaneous and chemically induced colorectal cancer through decreasing lipid synthesis and NADPH production [193]. The ACAT1 inhibitor AH also significantly induced GNPAT ubiquitination and degradation through targeting its acetylation, which markedly inhibited hepatocarcinogenesis in mice [139]. Sorafenib is a modestly effective multi-kinase inhibitor that has been approved for the treatment of advanced HCC. Sorafenib-induced tumor cell killing is mediated by the AMPK-mTOR-SREBP1 signaling pathway [172]. Mono-unsaturated fatty acids, such as oleic acid, are significantly reduced following sorafenib treatment. In HCC cells, sorafenib also inhibits ATP production, leading to phosphorylation-mediated AMPK activation, a reduction of SREBP1 levels and phosphorylation of mTOR [194]. Silibinin inhibited SREBP1 nuclear translocation via activating AMPK-mediated SREBP1 phosphorylation, and ultimately inhibiting cancer cell proliferation [195]. Based on the above results, targeting the posttranslational modification of lipid metabolism enzyme brought new opportunities for cancer therapy.

**Table 2:** Targeting posttranslational modification of cancer lipid metabolism enzymes for cancer treatment

Protein	Targeting PTMs	Drugs	Function	References
HMGCR	De-ubiquitination	GSK2643943A	To de-stabilize HMGCR	[133]
FXR	SUMOylation	SP/GA	To enhance FXR protein levels	[190–192]
ME1	Phosphorylation	Ulixertinib	To repress ME1 de-ubiquitination and promote it degradation	[193]
GNPAT	Acetylation	AH	Mediated GNPAT ubiquitination and degradation	[139]
SREBP1	Phosphorylation	Sorafenib	Reduction SREBP1 protein levels	[194]
SREBP1	Phosphorylation	Silibinin	To block SREBP1 nuclear translocation	[195]

## Conclusion

Lipid metabolism in cancer cells and the TME is complex and highly subject to PTMs of many of its key and/or rate-limiting enzymes. Understanding these modifications in greater detail is likely to provide reasonable target candidates for cancer treatments.

Recently, metabolomics and RNA-seq have been used to show that, in some cases, lipid metabolism can be used to assess the prognosis and risk factors for cancer patients, thereby providing a novel molecular diagnostic approach [196, 197]. Targeting lipid metabolism directly using agents such as statins has also demonstrated promising results [198, 199]. Metabolomics and protein “PTM-omics” potentially provide additional unbiased methods to assess lipid metabolism in cancers and could potentially also be used for diagnostic purposes. Cancers might potentially be classifiable into different stages based on their metabolomics profiles just as they currently are using transcriptomic-based approaches. This in turn could allow for more precise individualization of various therapies. More specific, tumor classification and therapeutic option selection might be achievable using lipid metabolomics profiling, thus allowing additional diagnostic therapeutic and prognostic personalization and refinement.

Future work will likely reveal additional levels of metabolic control. The study of PTMs that modulate enzymatic activity, particularly those that prove mutually antagonistic, is likely to provide therapies that, together with more traditional ones, will prove additive or synergistic.

## Data Availability

Not applicable; all information in this review can be found in the reference list.

## Abbreviations

ACAT1: Acetyl-CoA acetyltransferase 1
ACC: Acetyl-CoA carboxylase
ACLY: ATP-citrate synthase
ALOX15: Arachidonic lipoxygenase 15
AMPK: 5'-AMP-activated protein kinase
AR: Androgen receptor
ATP: Adenosine triphosphate
CAFs: Cancer-associated fibroblasts
CBX4: Chromobox4
CCRK: Cell cycle-associated kinase
CEBPB: CCAAT/enhancer-binding protein beta, C/EBP beta
COP1: E3 ubiquitin-protein ligase COP1
COX2: Cyclooxygenase-2
CPT1: Carnitine palmityl transferase 1
DC: Dendritic cells
DGAT1: Diacylglycerol O-acyltransferase 1
ECM: Extracellular matrix
FA: Fatty acids
FABPs: Fatty acid-binding proteins
FAO: Fatty acid oxidation
FASN: Fatty acid synthase
FATP2: Fatty acid transporter 2
FBXW7: F-box/WD repeat-containing protein 7
GNPAT: Dihydroxyacetone phosphate acyltransferase
HCC: Hepatocellular carcinoma
HCP5: Histocompatibility leukocyte antigen complex P5
HIF-1α: Hypoxia-inducible factor 1-alpha
HMGCR: HMG-CoA reductase
hnRNPA1: Heterogeneous nuclear ribonucleoprotein A1
HRD1: E3 ubiquitin-protein ligase HRD1
HSC: Hepatic stellate cells
ICIs: Immune checkpoint inhibitors
IDOL: E3 ubiquitin-protein ligase MYLIP
IFN-γ: Interferon-γ
IGF2BP1/IGF2BP3: Insulin-like growth factor-2 mRNB-binding proteins 1/3
IKK-beta: Inhibitor of nuclear factor kappa-B kinase subunit beta
IL-4: Interleukin-4
INKT: Invariant natural killer T cells
LATS1: Large tumor suppressor
LCFAs: Long-chain fatty acids
LDLR: Low-density lipoprotein receptor
LIHC: Liver hepatocellular carcinoma
LILRB: Leukocyte immunoglobulin-like receptor B
LILRB2: Leukocyte immunoglobulin-like receptor subfamily B member 2
LKB1: Liver kinase B1
LTA: Lipoteichoic acid
LXR: Liver X receptor
M-CSF: Macrophage colony-stimulating factor
MDR: Multidrug-resistant cancer cells
MDSC: Myeloid-derived suppressor cells
ME1: Malic enzyme 1
MSDC: Mesenchymal stem cell
NASH: Non-alcoholic steatohepatitis
NEK1: Serine/threonine-protein kinase Nek1
NPC: Nuclear pore complex
PCAF: P300/Calc-binding protein (CBP)-related factor
PCK: Phosphoenolpyruvate carboxy-kinase
PDA: Pancreatic ductal adenocarcinoma
PGAM5: Serine/threonine-protein phosphatase
PGE2: Prostaglandin E2
PI3K: Phosphatidylinositol 3-kinase
PPAR: Peroxisome proliferator-activated receptor
PPARGC1α: PPARG coactivator 1α
PRAD: Prostate adenocarcinoma
PRMTs: Protein arginine methyltransferases
PTM: Posttranslational modification
ROS: Reactive oxygen species
S1P: Sphingosin-1-phosphate
SAE1: SUMO-activating enzyme subunit 1
SAE2: SUMO-activating enzyme subunit 2
SAH: S-adenosylhomocysteine
SAM: S-adenosylmethionine
SASP: Senior-related secretion phenotype
SDMA: Symmetric di-methylated arginine
SENP1: SUMO-specific protease I
SHP1: Protein-tyrosine phosphatase SHP-1
SHP2: SH2-tyrosine phosphatase
SIRT6: NAD-dependent protein deacetylase Sirt6
spHK1: Sphingosine kinase 1
spHK2: Sphingosine kinase 2
SREBPs: Sterol-regulation-element-binding proteins
STAT6: Signal transducer and activator of transcription 6
TAMs: Tumor-associated macrophages
TDEs: Tumor-derived exosomes
TGF-β2: Transforming growth factor beta-2 proprotein
TIDCs: Tumor-infiltrating dendritic cells
TME: Tumor microenvironment
Treg cells: Regulatory T cells
TRIM21: E3 ubiquitin-protein ligase TRIM21
UBC9: SUMO-conjugating enzyme UBC9
UBXD: UBX domain-containing protein
USP30: Ubiquitin carboxyl-terminal hydrolase 30
USP7: Ubiquitin-specific protease 7
VLCAD: Very long-chain acyl coenzyme A dehydrogenase
VLCFAs: Very long-chain fatty acids
ZNF451: E3 SUMO-protein ligase ZNF451

## References

[1] Hanahan D, Weinberg RA (2011). Hallmarks of cancer: the next generation. Cell.

[2] DeBerardinis RJ, Chandel NS (2016). Fundamentals of cancer metabolism. Sci Adv.

[3] Cheng C, Geng F, Cheng X, Guo D (2018). Lipid metabolism reprogramming and its potential targets in cancer. Cancer Commun (Lond).

[4] Xiao X, Tang JJ, Peng C (2017). Cholesterol modification of smoothened is required for hedgehog signaling. Mol Cell.

[5] Luo J, Yang H, Song BL (2020). Mechanisms and regulation of cholesterol homeostasis. Nat Rev Mol Cell Biol.

[6] Gong J, Lin Y, Zhang H (2020). Reprogramming of lipid metabolism in cancer-associated fibroblasts potentiates migration of colorectal cancer cells. Cell Death Dis.

[7] Kim D, Wu Y, Li Q, Oh YK (2021). Nanoparticle-mediated lipid metabolic reprogramming of T cells in tumor microenvironments for immunometabolic therapy. Nanomicro Lett.

[8] Maan M, Peters JM, Dutta M, Patterson AD (2018). Lipid metabolism and lipophagy in cancer. Biochem Biophys Res Commun.

[9] Alannan M, Fayyad-Kazan H, Trezeguet V, Merched A (2020). Targeting lipid metabolism in liver cancer. Biochemistry.

[10] Bort A, Sanchez BG, de Miguel I, Mateos-Gomez PA, Diaz-Laviada I (2020). Dysregulated lipid metabolism in hepatocellular carcinoma cancer stem cells. Mol Biol Rep.

[11] Rohrig F, Schulze A (2016). The multifaceted roles of fatty acid synthesis in cancer. Nat Rev Cancer.

[12] Koundouros N, Poulogiannis G (2020). Reprogramming of fatty acid metabolism in cancer. Br J Cancer.

[13] Ping Q, Yan R, Cheng X (2021). Cancer-associated fibroblasts: overview, progress, challenges, and directions. Cancer Gene Ther.

[14] Yu W, Lei Q, Yang L (2021). Contradictory roles of lipid metabolism in immune response within the tumor microenvironment. J Hematol Oncol.

[15] Hu A, Zhao XT, Tu H (2018). PIP4K2A regulates intracellular cholesterol transport through modulating PI(4,5)P2 homeostasis. J Lipid Res.

[16] Xiao J, Luo J, Hu A (2019). Cholesterol transport through the peroxisome-ER membrane contacts tethered by PI(4,5)P2 and extended synaptotagmins. Sci China Life Sci.

[17] Prochownik EV, Wang H (2021). The metabolic fates of pyruvate in normal and neoplastic cells. Cells.

[18] Bose S, Ramesh V, Locasale JW (2019). Acetate metabolism in physiology, cancer, and beyond. Trends Cell Biol.

[19] Calhoun S, Duan L, Maki CG (2022). Acetyl-CoA synthetases ACSS1 and ACSS2 are 4-hydroxytamoxifen responsive factors that promote survival in tamoxifen treated and estrogen deprived cells. Transl Oncol.

[20] Cheng C, Geng F, Li Z (2022). Ammonia stimulates SCAP/Insig dissociation and SREBP-1 activation to promote lipogenesis and tumour growth. Nat Metab.

[21] Jones SF, Infante JR (2015). Molecular pathways: fatty acid synthase. Clin Cancer Res.

[22] Shimano H, Sato R (2017). SREBP-regulated lipid metabolism: convergent physiology—divergent pathophysiology. Nat Rev Endocrinol.

[23] Cheng C, Ru P, Geng F (2015). Glucose-mediated N-glycosylation of SCAP is essential for SREBP-1 activation and tumor growth. Cancer Cell.

[24] Granchi C (2018). ATP citrate lyase (ACLY) inhibitors: An anti-cancer strategy at the crossroads of glucose and lipid metabolism. Eur J Med Chem.

[25] Menendez JA, Lupu R (2017). Fatty acid synthase (FASN) as a therapeutic target in breast cancer. Expert Opin Ther Targets.

[26] Enciu AM, Radu E, Popescu ID, Hinescu ME, Ceafalan LC (2018). Targeting CD36 as biomarker for metastasis prognostic: how far from translation into clinical practice?. Biomed Res Int.

[27] Go GW, Mani A (2012). Low-density lipoprotein receptor (LDLR) family orchestrates cholesterol homeostasis. Yale J Biol Med.

[28] Rogers MA, Liu J, Song BL (2015). Acyl-CoA:cholesterol acyltransferases (ACATs/SOATs): enzymes with multiple sterols as substrates and as activators. J Steroid Biochem.

[29] Bovenga F, Sabba C, Moschetta A (2015). Uncoupling nuclear receptor LXR and cholesterol metabolism in cancer. Cell Metab.

[30] Phillips MC (2018). Is ABCA1 a lipid transfer protein?. J Lipid Res.

[31] Chistiakov DA, Bobryshev YV, Orekhov AN (2016). Macrophage-mediated cholesterol handling in atherosclerosis. J Cell Mol Med.

[32] Kemmerer M, Wittig I, Richter F, Brune B, Namgaladze D (2016). AMPK activates LXRalpha and ABCA1 expression in human macrophages. Int J Biochem Cell Biol.

[33] Bougarne N, Weyers B, Desmet SJ (2018). Molecular actions of PPARalpha in lipid metabolism and inflammation. Endocr Rev.

[34] Pawlak M, Lefebvre P, Staels B (2015). Molecular mechanism of PPARalpha action and its impact on lipid metabolism, inflammation and fibrosis in non-alcoholic fatty liver disease. J Hepatol.

[35] Schlaepfer IR, Joshi M (2020). CPT1A-mediated fat oxidation, mechanisms, and therapeutic potential. Endocrinology.

[36] Santos CR, Schulze A (2012). Lipid metabolism in cancer. FEBS J.

[37] Yi M, Li J, Chen S (2018). Emerging role of lipid metabolism alterations in Cancer stem cells. J Exp Clin Cancer Res.

[38] Fernandez LP, de Gomez CM, de Ramirez MA (2020). Alterations of lipid metabolism in cancer: implications in prognosis and treatment. Front Oncol.

[39] Luo X, Cheng C, Tan Z (2017). Emerging roles of lipid metabolism in cancer metastasis. Mol Cancer.

[40] Vriens K, Christen S, Parik S (2019). Evidence for an alternative fatty acid desaturation pathway increasing cancer plasticity. Nature.

[41] Chen L, Chen XW, Huang X (2019). Regulation of glucose and lipid metabolism in health and disease. Sci China Life Sci.

[42] Pyne NJ, El Buri A, Adams DR, Pyne S (2018). Sphingosine 1-phosphate and cancer. Adv Biol Regul.

[43] Schneider G (2020). S1P signaling in the tumor microenvironment. Adv Exp Med Biol.

[44] Sukocheva OA, Furuya H, Ng ML (2020). Sphingosine kinase and sphingosine-1-phosphate receptor signaling pathway in inflammatory gastrointestinal disease and cancers: a novel therapeutic target. Pharmacol Ther.

[45] Nganga R, Oleinik N, Ogretmen B (2018). Mechanisms of ceramide-dependent cancer cell death. Adv Cancer Res.

[46] Gomez-Larrauri A, Presa N, Dominguez-Herrera A (2020). Role of bioactive sphingolipids in physiology and pathology. Essays Biochem.

[47] Laplane L, Duluc D, Bikfalvi A, Larmonier N, Pradeu T (2019). Beyond the tumour microenvironment. Int J Cancer.

[48] Weatherill AR, Lee JY, Zhao L (2005). Saturated and polyunsaturated fatty acids reciprocally modulate dendritic cell functions mediated through TLR4. J Immunol.

[49] Peng X, He Y, Huang J, Tao Y, Liu S (2021). Metabolism of dendritic cells in tumor microenvironment: for immunotherapy. Front Immunol.

[50] Ma XZ, Xiao LL, Liu LT (2021). CD36-mediated ferroptosis dampens intratumoral CD8(+) T cell effector function and impairs their antitumor ability. Cell Metab.

[51] Yang W, Bai Y, Xiong Y (2016). Potentiating the antitumour response of CD8(+) T cells by modulating cholesterol metabolism. Nature.

[52] Kishton RJ, Barnes CE, Nichols AG (2016). AMPK Is Essential to balance glycolysis and mitochondrial metabolism to control T-all cell stress and survival. Cell Metab.

[53] Zhang Q, Wang H, Mao C (2018). Fatty acid oxidation contributes to IL-1beta secretion in M2 macrophages and promotes macrophage-mediated tumor cell migration. Mol Immunol.

[54] Zhao F, Xiao C, Evans KS (2018). Paracrine Wnt5a-beta-catenin signaling triggers a metabolic program that drives dendritic cell tolerization. Immunity.

[55] Wang Y, Wang Y, Ren Y (2022). Metabolic modulation of immune checkpoints and novel therapeutic strategies in cancer. Semin Cancer Biol.

[56] Wu T, Dai Y (2017). Tumor microenvironment and therapeutic response. Cancer Lett.

[57] Arneth B (2019). Tumor Microenvironment. Medicina.

[58] Trempolec N, Degavre C, Doix B (2020). Acidosis-induced TGF-beta2 production promotes lipid droplet formation in dendritic cells and alters their Potential to support anti-mesothelioma T cell response. Cancers.

[59] Otto AM (2020). Metabolic constants and plasticity of cancer cells in a limiting glucose and glutamine microenvironment-a pyruvate perspective. Front Oncol.

[60] Corbet C, Bastien E, de Santiago Jesus JP (2020). TGFbeta2-induced formation of lipid droplets supports acidosis-driven EMT and the metastatic spreading of cancer cells. Nat Commun.

[61] Hao Y, Li D, Xu Y (2019). Investigation of lipid metabolism dysregulation and the effects on immune microenvironments in pan-cancer using multiple omics data. BMC Bioinform.

[62] Li X, Wenes M, Romero P (2019). Navigating metabolic pathways to enhance antitumour immunity and immunotherapy. Nat Rev Clin Oncol.

[63] Corn KC, Windham MA, Rafat M (2020). Lipids in the tumor microenvironment: from cancer progression to treatment. Prog Lipid Res.

[64] Petty AJ, Yang Y (2017). Tumor-associated macrophages: implications in cancer immunotherapy. Immunotherapy.

[65] Su P, Wang Q, Bi E (2020). Enhanced lipid accumulation and metabolism are required for the differentiation and activation of tumor-associated macrophages. Cancer Res.

[66] Ligorio M, Sil S, Malagon-Lopez J (2019). Stromal microenvironment shapes the intratumoral architecture of pancreatic cancer. Cell.

[67] Zhang H, Deng T, Liu R (2020). CAF secreted miR-522 suppresses ferroptosis and promotes acquired chemo-resistance in gastric cancer. Mol Cancer.

[68] Wu H, Liu B, Chen Z, Li G, Zhang Z (2020). MSC-induced lncRNA HCP5 drove fatty acid oxidation through miR-3619-5p/AMPK/PGC1alpha/CEBPB axis to promote stemness and chemo-resistance of gastric cancer. Cell Death Dis.

[69] Yin X, Zeng W, Wu B (2020). PPARalpha inhibition overcomes tumor-derived exosomal lipid-induced dendritic cell dysfunction. Cell Rep.

[70] Li I, Nabet BY (2019). Exosomes in the tumor microenvironment as mediators of cancer therapy resistance. Mol Cancer.

[71] Manzo T, Prentice BM, Anderson KG (2020). Accumulation of long-chain fatty acids in the tumor microenvironment drives dysfunction in intrapancreatic CD8+ T cells. J Exp Med.

[72] Tang R, Xu J, Zhang B (2020). Ferroptosis, necroptosis, and pyroptosis in anticancer immunity. J Hematol Oncol.

[73] Wang W, Green M, Choi JE (2019). CD8(+) T cells regulate tumour ferroptosis during cancer immunotherapy. Nature.

[74] Ecker C, Guo L, Voicu S (2018). Differential reliance on lipid metabolism as a salvage pathway underlies functional differences of T cell subsets in poor nutrient environments. Cell Rep.

[75] Datta M, Coussens LM, Nishikawa H, Hodi FS, Jain RK (2019). Reprogramming the tumor microenvironment to improve immunotherapy: emerging strategies and combination therapies. Am Soc Clin Oncol Educ Book.

[76] Cluxton D, Petrasca A, Moran B, Fletcher JM (2019). Differential regulation of human Treg and Th17 cells by fatty acid synthesis and glycolysis. Front Immunol.

[77] Lim SA, Wei J, Nguyen TM (2021). Lipid signalling enforces functional specialization of Treg cells in tumours. Nature.

[78] Kidani Y, Elsaesser H, Hock MB (2013). Sterol regulatory element-binding proteins are essential for the metabolic programming of effector T cells and adaptive immunity. Nat Immunol.

[79] Sasidharan Nair V, Elkord E (2018). Immune checkpoint inhibitors in cancer therapy: a focus on T-regulatory cells. Immunol Cell Biol.

[80] Lee JG, Jaeger KE, Seki Y (2021). Human CD36(hi) monocytes induce Foxp3(+) CD25(+) T cells with regulatory functions from CD4 and CD8 subsets. Immunology.

[81] Funaoka H, Kanda T, Fujii H (2010). Intestinal fatty acid-binding protein (I-FABP) as a new biomarker for intestinal diseases. Rinsho Byori Jpn J Clin Pathol.

[82] Field CS, Baixauli F, Kyle RL (2020). Mitochondrial integrity regulated by lipid metabolism is a cell-intrinsic checkpoint for Treg suppressive function. Cell Metab.

[83] Wu B, Qiu J, Zhao TV (2020). Succinyl-CoA ligase deficiency in pro-inflammatory and tissue-invasive T cells. Cell Metab.

[84] Imbert C, Montfort A, Fraisse M (2020). Resistance of melanoma to immune checkpoint inhibitors is overcome by targeting the sphingosine kinase-1. Nat Commun.

[85] Wei J, Zheng W, Chapman NM, Geiger TL, Chi H (2021). T cell metabolism in homeostasis and cancer immunity. Curr Opin Biotechnol.

[86] Liu C, Chikina M, Deshpande R (2019). Treg cells promote the SREBP1-dependent metabolic fitness of tumor-promoting macrophages via repression of CD8(+) T cell-derived interferon-gamma. Immunity.

[87] Pineiro Fernandez J, Luddy KA, Harmon C, O'Farrelly C (2019). Hepatic tumor microenvironments and effects on NK cell phenotype and function. Int J Mol Sci.

[88] Fu S, He K, Tian C (2020). Impaired lipid biosynthesis hinders anti-tumor efficacy of intratumoral iNKT cells. Nat Commun.

[89] Goossens P, Rodriguez-Vita J, Etzerodt A (2019). Membrane cholesterol efflux drives tumor-associated macrophage reprogramming and tumor progression. Cell Metab.

[90] Chen HM, van der Touw W, Wang YS (2018). Blocking immunoinhibitory receptor LILRB2 reprograms tumor-associated myeloid cells and promotes antitumor immunity. J Clin Invest.

[91] Singh L, Muise ES, Bhattacharya A (2021). ILT3 (LILRB4) promotes the immunosuppressive function of tumor-educated human monocytic myeloid-derived suppressor cells. Mol Cancer Res.

[92] Veglia F, Tyurin VA, Blasi M (2019). Fatty acid transport protein 2 reprograms neutrophils in cancer. Nature.

[93] Bonaventura P, Shekarian T, Alcazer V (2019). Cold Tumors: A Therapeutic Challenge for Immunotherapy. Front Immunol.

[94] Hinshaw DC, Shevde LA (2019). The tumor microenvironment innately modulates cancer progression. Cancer Res.

[95] Tao L, Zhu F, Xu F (2015). Co-targeting cancer drug escape pathways confers clinical advantage for multi-target anticancer drugs. Pharmacol Res.

[96] Liu X, Liu R, Bai Y (2020). Post-translational modifications of protein in response to ionizing radiation. Cell Biochem Funct.

[97] Tolsma TO, Hansen JC (2019). Post-translational modifications and chromatin dynamics. Essays Biochem.

[98] Czuba LC, Hillgren KM, Swaan PW (2018). Post-translational modifications of transporters. Pharmacol Ther.

[99] Silva AMN, Vitorino R, Domingues MRM, Spickett CM, Domingues P (2013). Post-translational modifications and mass spectrometry detection. Free Radic Biol Med.

[100] Ke M, Shen H, Wang L (2016). Identification, quantification, and site localization of protein posttranslational modifications via mass spectrometry-based proteomics. Adv Exp Med Biol.

[101] Elguero B, Gonilski Pacin D, Cárdenas Figueroa C, Fuertes M, Arzt E (2019). Modifications in the cellular proteome and their clinical application. Medicina (B Aires).

[102] Chatterjee B, Thakur SS (2018). Investigation of post-translational modifications in type 2 diabetes. Clin Proteomics.

[103] Melo-Braga MN, Ibanez-Vea M, Kulej K, Larsen MR (2021). Comprehensive protocol to simultaneously study protein phosphorylation, acetylation, and N-linked sialylated glycosylation. Methods Mol Biol.

[104] Beltrao P, Bork P, Krogan NJ, van Noort V (2013). Evolution and functional cross-talk of protein post-translational modifications. Mol Syst Biol.

[105] Wang Z, Yu T, Huang P (2016). Post-translational modifications of FOXO family proteins (Review). Mol Med Rep.

[106] Sikarwar AS, Bhagirath AY, Dakshinamurti S (2019). Effects of post-translational modifications on membrane localization and signaling of prostanoid GPCR-G protein complexes and the role of hypoxia. J Membr Biol.

[107] Han ZJ, Feng YH, Gu BH, Li YM, Chen H (2018). The post-translational modification, SUMOylation, and cancer (Review). Int J Oncol.

[108] Heo K-S (2019). Regulation of post-translational modification in breast cancer treatment. BMB Rep.

[109] Wu Z, Huang R, Yuan L (2019). Crosstalk of intracellular post-translational modifications in cancer. Arch Biochem Biophys.

[110] Chang PC, Campbell M, Robertson ES (2016). Human oncogenic herpesvirus and post-translational modifications - phosphorylation and SUMOylation. Front Microbiol.

[111] Ooshima A, Park J, Kim SJ (2019). Phosphorylation status at Smad3 linker region modulates transforming growth factor-beta-induced epithelial-mesenchymal transition and cancer progression. Cancer Sci.

[112] Watanabe N, Osada H (2012). Phosphorylation-dependent protein-protein interaction modules as potential molecular targets for cancer therapy. Curr Drug Targets.

[113] Niemi NM, MacKeigan JP (2013). Mitochondrial phosphorylation in apoptosis: flipping the death switch. Antioxid Redox Signal.

[114] Humphrey SJ, James DE, Mann M (2015). Protein phosphorylation: a major switch mechanism for metabolic regulation. Trends Endocrinol Metab.

[115] Carling D (2017). AMPK signalling in health and disease. Curr Opin Cell Biol.

[116] Anbalagan M, Rowan BG (2015). Estrogen receptor alpha phosphorylation and its functional impact in human breast cancer. Mol Cell Endocrinol.

[117] Kastrati I, Semina S, Gordon B, Smart E (2019). Insights into how phosphorylation of estrogen receptor at serine 305 modulates tamoxifen activity in breast cancer. Mol Cell Endocrinol.

[118] Mevizou R, Sirvent A, Roche S (2019). Control of tyrosine kinase signalling by small adaptors in colorectal cancer. Cancers.

[119] Tomasi ML, Ramani K (2018). SUMOylation and phosphorylation cross-talk in hepatocellular carcinoma. Transl Gastroenterol Hepatol.

[120] Ubersax JA, Ferrell JE (2007). Mechanisms of specificity in protein phosphorylation. Nat Rev Mol Cell Biol.

[121] Hanafusa H, Garber EA, Hanafusa T (1986). Transformation by p60src with altered N-terminal sequences. Princess Takamatsu Symp.

[122] Singh V, Ram M, Kumar R (2017). Phosphorylation: implications in cancer. Protein J.

[123] Ren Y, Shen HM (2019). Critical role of AMPK in redox regulation under glucose starvation. Redox Biol.

[124] Strickland M, Stoll EA (2017). Metabolic Reprogramming in glioma. Front Cell Dev Biol.

[125] Li Y, Xu S, Mihaylova MM (2011). AMPK phosphorylates and inhibits SREBP activity to attenuate hepatic steatosis and atherosclerosis in diet-induced insulin-resistant mice. Cell Metab.

[126] Bakan I, Laplante M (2012). Connecting mTORC1 signaling to SREBP-1 activation. Curr Opin Lipidol.

[127] Aoki M, Fujishita T (2017). Oncogenic roles of the PI3K/AKT/mTOR Axis. Curr Top Microbiol Immunol.

[128] Currie E, Schulze A, Zechner R, Walther TC, Farese RV (2013). Cellular fatty acid metabolism and cancer. Cell Metab.

[129] Lee MS, Kim KJ, Kim D, Lee KE, Hwang JK (2011). meso-Dihydroguaiaretic acid inhibits hepatic lipid accumulation by activating AMP-activated protein kinase in human HepG2 cells. Biol Pharm Bull.

[130] O'Neill HM, Lally JS, Galic S (2014). AMPK phosphorylation of ACC2 is required for skeletal muscle fatty acid oxidation and insulin sensitivity in mice. Diabetologia.

[131] Rios Garcia M, Steinbauer B, Srivastava K (2017). Acetyl-CoA carboxylase 1-dependent protein acetylation controls breast cancer metastasis and recurrence. Cell Metab.

[132] Gu L, Zhu Y, Lin X (2021). The IKKbeta-USP30-ACLY axis controls lipogenesis and tumorigenesis. Hepatology.

[133] Lu XY, Shi XJ, Hu A (2020). Feeding induces cholesterol biosynthesis via the mTORC1-USP20-HMGCR axis. Nature.

[134] Zhang X, Song Y, Feng M (2015). Thyroid-stimulating hormone decreases HMG-CoA reductase phosphorylation via AMP-activated protein kinase in the liver. J Lipid Res.

[135] Gao T, Li M, Mu G (2019). PKCzeta phosphorylates SIRT6 to mediate fatty acid beta-oxidation in colon cancer cells. Neoplasia.

[136] Menzies KJ, Zhang H, Katsyuba E, Auwerx J (2016). Protein acetylation in metabolism - metabolites and cofactors. Nat Rev Endocrinol.

[137] Lin SY, Li TY, Liu Q (2012). GSK3-TIP60-ULK1 signaling pathway links growth factor deprivation to autophagy. Science.

[138] Li TY, Song L, Sun Y (2018). Tip60-mediated lipin 1 acetylation and ER translocation determine triacylglycerol synthesis rate. Nat Commun.

[139] Gu L, Zhu Y, Lin X (2020). Stabilization of FASN by ACAT1-mediated GNPAT acetylation promotes lipid metabolism and hepatocarcinogenesis. Oncogene.

[140] Song NY, Na HK, Baek JH, Surh YJ (2014). Docosahexaenoic acid inhibits insulin-induced activation of sterol regulatory-element binding protein 1 and cyclooxygenase-2 expression through upregulation of SIRT1 in human colon epithelial cells. Biochem Pharmacol.

[141] Liu L, Xie B, Fan M (2020). Low-level saturated fatty acid palmitate benefits liver cells by boosting mitochondrial metabolism via CDK1-SIRT3-CPT2 cascade. Dev Cell.

[142] Li M, Hong W, Hao C (2017). Hepatic stellate cell-specific deletion of SIRT1 exacerbates liver fibrosis in mice. Biochim Biophys Acta Mol Basis Dis.

[143] Deng L, Meng T, Chen L, Wei W, Wang P (2020). The role of ubiquitination in tumorigenesis and targeted drug discovery. Signal Transduct Target Ther.

[144] Ducker C, Shaw PE (2021). USP17-mediated de-ubiquitination and cancer: clients cluster around the cell cycle. Int J Biochem Cell Biol.

[145] Ramakrishna S, Suresh B, Baek KH (2011). The role of deubiquitinating enzymes in apoptosis. Cell Mol Life Sci.

[146] Lim KH, Song MH, Baek KH (2016). Decision for cell fate: deubiquitinating enzymes in cell cycle checkpoint. Cell Mol Life Sci.

[147] Qi L, Heredia JE, Altarejos JY (2006). TRB3 links the E3 ubiquitin ligase COP1 to lipid metabolism. Science.

[148] Yu J, Deng R, Zhu HH (2013). Modulation of fatty acid synthase degradation by concerted action of p38 MAP kinase, E3 ligase COP1, and SH2-tyrosine phosphatase Shp2. J Biol Chem.

[149] Calvisi DF, Wang C, Ho C (2011). Increased lipogenesis, induced by AKT-mTORC1-RPS6 signaling, promotes development of human hepatocellular carcinoma. Gastroenterology.

[150] Menzies SA, Volkmar N, van den Boomen DJ (2018). The sterol-responsive RNF145 E3 ubiquitin ligase mediates the degradation of HMG-CoA reductase together with gp78 and Hrd1. Elife.

[151] Jiang LY, Jiang W, Tian N (2018). Ring finger protein 145 (RNF145) is a ubiquitin ligase for sterol-induced degradation of HMG-CoA reductase. J Biol Chem.

[152] Loregger A, Raaben M, Tan J (2017). Haploid mammalian genetic screen identifies UBXD8 as a key determinant of HMGCR degradation and cholesterol biosynthesis. Arterioscler Thromb Vasc Biol.

[153] Hwang S, Nguyen AD, Jo Y (2017). Hypoxia-inducible factor 1alpha activates insulin-induced gene 2 (Insig-2) transcription for degradation of 3-hydroxy-3-methylglutaryl (HMG)-CoA reductase in the liver. J Biol Chem.

[154] Gelsomino G, Corsetto PA, Campia I (2013). Omega 3 fatty acids chemosensitize multidrug resistant colon cancer cells by down-regulating cholesterol synthesis and altering detergent resistant membranes composition. Mol Cancer.

[155] Stevenson J, Luu W, Kristiana I, Brown AJ (2014). Squalene mono-oxygenase, a key enzyme in cholesterol synthesis, is stabilized by unsaturated fatty acids. Biochem J.

[156] Foresti O, Ruggiano A, Hannibal-Bach HK, Ejsing CS, Carvalho P (2013). Sterol homeostasis requires regulated degradation of squalene monooxygenase by the ubiquitin ligase Doa10/Teb4. Elife.

[157] Zelcer N, Hong C, Boyadjian R, Tontonoz P (2009). LXR regulates cholesterol uptake through Idol-dependent ubiquitination of the LDL receptor. Science.

[158] Wilson VG (2017). Introduction to sumoylation. Adv Exp Med Biol.

[159] Huang CJ, Wu D, Khan FA, Huo LJ (2015). DeSUMOylation: an important therapeutic target and protein regulatory event. DNA Cell Biol.

[160] Yang ST, Yen CJ, Lai CH (2013). SUMOylated CPAP is required for IKK-mediated NF-kappaB activation and enhances HBx-induced NF-kappaB signaling in HCC. J Hepatol.

[161] Mei L, Yuan L, Shi W (2017). SUMOylation of large tumor suppressor 1 at Lys751 attenuates its kinase activity and tumor-suppressor functions. Cancer Lett.

[162] Zubiete-Franco I, Garcia-Rodriguez JL, Lopitz-Otsoa F (2019). SUMOylation regulates LKB1 localization and its oncogenic activity in liver cancer. EBioMedicine.

[163] Li J, Xu Y, Long XD (2014). Cbx4 governs HIF-1alpha to potentiate angiogenesis of hepatocellular carcinoma by its SUMO E3 ligase activity. Cancer Cell.

[164] Cui CP, Wong CC, Kai AK (2017). SENP1 promotes hypoxia-induced cancer stemness by HIF-1alpha deSUMOylation and SENP1/HIF-1alpha positive feedback loop. Gut.

[165] Choi HK, Choi KC, Yoo JY (2011). Reversible SUMOylation of TBL1-TBLR1 regulates beta-catenin-mediated Wnt signaling. Mol Cell.

[166] Du JX, Bialkowska AB, McConnell BB, Yang VW (2008). SUMOylation regulates nuclear localization of Kruppel-like factor 5. J Biol Chem.

[167] Peng C, Wong CC (2017). The story of protein arginine methylation: characterization, regulation, and function. Expert Rev Proteomics.

[168] vanLieshout TL, Ljubicic V (2019). The emergence of protein arginine methyltransferases in skeletal muscle and metabolic disease. Am J Physiol Endocrinol Metab.

[169] Al-Hamashi AA, Diaz K, Huang R (2020). Non-Histone arginine methylation by protein arginine methyltransferases. Curr Protein Pept Sci.

[170] Liu L, Zhao X, Zhao L (2016). Arginine methylation of SREBP1a via PRMT5 promotes de novo lipogenesis and tumor growth. Cancer Res.

[171] Zhu Y, Gu L, Lin X (2020). Dynamic regulation of ME1 phosphorylation and acetylation affects lipid metabolism and colorectal tumorigenesis. Mol Cell.

[172] Caron C, Boyault C, Khochbin S (2005). Regulatory cross-talk between lysine acetylation and ubiquitination: role in the control of protein stability. BioEssays.

[173] Lin R, Tao R, Gao X (2013). Acetylation stabilizes ATP-citrate lyase to promote lipid biosynthesis and tumor growth. Mol Cell.

[174] Bengoechea-Alonso MT, Ericsson J (2016). The phosphorylation-dependent regulation of nuclear SREBP1 during mitosis links lipid metabolism and cell growth. Cell Cycle.

[175] Sundqvist A, Bengoechea-Alonso MT, Ye X (2005). Control of lipid metabolism by phosphorylation-dependent degradation of the SREBP family of transcription factors by SCF(Fbw7). Cell Metab.

[176] Lin HP, Cheng ZL, He RY (2016). Destabilization of fatty acid synthase by acetylation inhibits de novo lipogenesis and tumor cell growth. Cancer Res.

[177] Butler LM, Perone Y, Dehairs J (2020). Lipids and cancer: emerging roles in pathogenesis, diagnosis and therapeutic intervention. Adv Drug Deliv Rev.

[178] Pemble CW, Johnson LC, Kridel SJ, Lowther WT (2007). Crystal structure of the thioesterase domain of human fatty acid synthase inhibited by Orlistat. Nat Struct Mol Biol.

[179] Munir R, Lisec J, Swinnen JV, Zaidi N (2022). Too complex to fail? Targeting fatty acid metabolism for cancer therapy. Prog Lipid Res.

[180] Zhu Y, Gu L, Lin X (2022). P53 deficiency affects cholesterol esterification to exacerbate hepatocarcinogenesis. Hepatology.

[181] Zhu Y, Gu L, Lin X (2022). Ceramide-mediated gut dysbiosis enhances cholesterol esterification and promotes colorectal tumorigenesis in mice. JCI Insight.

[182] Riscal R, Skuli N, Simon MC (2019). Even cancer cells watch their cholesterol!. Mol Cell.

[183] Liu D, Wong CC, Fu L (2018). Squalene epoxidase drives NAFLD-induced hepatocellular carcinoma and is a pharmaceutical target. Sci Transl Med.

[184] Jump DB, Torres-Gonzalez M, Olson LK (2011). Soraphen A, an inhibitor of acetyl CoA carboxylase activity, interferes with fatty acid elongation. Biochem Pharmacol.

[185] Beckers A, Organe S, Timmermans L (2007). Chemical inhibition of acetyl-CoA carboxylase induces growth arrest and cytotoxicity selectively in cancer cells. Cancer Res.

[186] Nath A, Li I, Roberts LR, Chan C (2015). Elevated free fatty acid uptake via CD36 promotes epithelial-mesenchymal transition in hepatocellular carcinoma. Sci Rep.

[187] Brovkovych V, Izhar Y, Danes JM (2018). Fatostatin induces pro- and anti-apoptotic lipid accumulation in breast cancer. Oncogenesis.

[188] Li N, Zhou ZS, Shen Y (2017). Inhibition of the sterol regulatory element-binding protein pathway suppresses hepatocellular carcinoma by repressing inflammation in mice. Hepatology.

[189] Marengo A, Rosso C, Bugianesi E (2016). Liver cancer: connections with obesity, fatty liver, and cirrhosis. Annu Rev Med.

[190] Zhou J, Cui S, He Q (2020). SUMOylation inhibitors synergize with FXR agonists in combating liver fibrosis. Nat Commun.

[191] Bilodeau S, Caron V, Gagnon J, Kuftedjian A, Tremblay A (2017). A CK2-RNF4 interplay coordinates non-canonical SUMOylation and degradation of nuclear receptor FXR. J Mol Cell Biol.

[192] Kim DH, Xiao Z, Kwon S (2015). A dysregulated acetyl/SUMO switch of FXR promotes hepatic inflammation in obesity. EMBO J.

[193] Zhu Y, Gu L, Lin X (2021). USP19 exacerbates lipogenesis and colorectal carcinogenesis by stabilizing ME1. Cell Rep.

[194] Zhang Q, He Y, Luo N (2019). Landscape and dynamics of single immune cells in hepatocellular carcinoma. Cell.

[195] Nambiar DK, Deep G, Singh RP, Agarwal C, Agarwal R (2014). Silibinin inhibits aberrant lipid metabolism, proliferation and emergence of androgen-independence in prostate cancer cells via primarily targeting the sterol response element binding protein 1. Oncotarget.

[196] Zheng M, Mullikin H, Hester A (2020). Development and validation of a novel 11-gene prognostic model for serous ovarian carcinomas based on lipid metabolism expression profile. Int J Mol Sci.

[197] Chaudhry S, Thomas SN, Simmons GE (2022). Targeting lipid metabolism in the treatment of ovarian cancer. Oncotarget.

[198] Chen RR, Yung MMH, Xuan Y (2019). Targeting of lipid metabolism with a metabolic inhibitor cocktail eradicates peritoneal metastases in ovarian cancer cells. Commun Biol.

[199] Pyragius CE, Fuller M, Ricciardelli C, Oehler MK (2013). Aberrant lipid metabolism: an emerging diagnostic and therapeutic target in ovarian cancer. Int J Mol Sci.

